# Sphingosine Kinase 2 Inhibition Ameliorates Neuroinflammation in Diet-Induced Obese Mice

**DOI:** 10.14336/AD.2025.0636

**Published:** 2026-09-03

**Authors:** Lotte Vanherle, Cecilia Skoug, Lisa Teresa Porschen, Fátima Gimeno-Ferrer, Frank Matthes, João M. N. Duarte, Anja Meissner

**Affiliations:** ^1^Department of Experimental Medical Science, Faculty of Medicine, Lund University, 221 84 Lund, Sweden.; ^2^Wallenberg Center for Molecular Medicine, Faculty of Medicine, Lund University, 221 84 Lund, Sweden.; ^3^Division of Physiology & Vascular Biology, Institute of Theoretical Medicine, Faculty of Medicine, University of Augsburg, Augsburg, Germany.

**Keywords:** Sphingosine-1-phosphate, SphK2 inhibitor, neuroinflammation, obesity

## Abstract

Obesity is associated with low-grade inflammation and microgliosis, contributing to brain dysfunction and cognitive decline. Sphingosine-1-phosphate (S1P) and its generating enzymes sphingosine kinase 1/2 (SphK1/2) have been implicated in metabolic and inflammatory regulation. Plasma S1P levels are elevated in obese mice and humans, and genetic ablation of SphK2 in mice protects from age- and diet-induced weight gain. As SphK2 is the predominant isoform in the brain, the present study examined whether pharmacological SphK2 inhibition mitigates obesity-associated microgliosis. Male C57BL/6J mice were fed either a high-fat diet (HFD; 60% fat) or control diet (CD; 10% fat) for 9 weeks. After 7 weeks, mice received the SphK2 inhibitor ABC294640 (SphK2i; 5 mg/kg, s.c.) or vehicle every other day for two weeks. Brain tissue was analyzed for microglial morphology (classification of ramified, intermediate, and amoeboid phenotypes, as well as branch length and endpoints), profiling of pro-inflammatory cytokines and S1P pathway components, and S1P concentrations. SphK2 inhibition attenuated HFD-induced microgliosis in cortex and dentate gyrus, with partial restoration of microglial arborization and branch length in cortex, in the absence of pro-inflammatory cytokine expression alterations. S1P pathway responses showed regional specificity, including elevated cortical S1P levels after HFD feeding accompanied by reduced S1P receptor 1 (S1PR1) expression, whereas hippocampal S1P levels and S1PR1 expression remained unchanged. SphK2 was undetectable in cortical microglia, while hippocampal microglia were SphK2-positive. Despite regional specificities of HFD-induced S1P/S1PR1 alterations, pharmacological SphK2 inhibition reduced microglial activation across regions, highlighting its potential relevance for obesity-associated neuroinflammation in a brain region-specific manner.

## INTRODUCTION

Obesity and metabolic syndrome resulting from overconsumption of fat-rich dietary products are not only risk factors for cardiovascular and metabolic disorders [1], but they also contribute to cognitive decline and neurodegenerative diseases such as Alzheimer’s disease [2] and Parkinson’s disease [3]. A growing body of evidence suggests that obesity-induced gliosis and neuroinflammation are key drivers to these neurological complications, with microglia as the resident immune cells of the central nervous system (CNS) playing a central role [4-6]. Under obesogenic conditions, microglia exhibit morphological and functional changes and adopt a pro-inflammatory phenotype, potentially disrupting synaptic function and promoting neuronal injury [7-9]. Notably, while overt cytokine overexpression in cortex and hippocampus is often subtle in diet-induced models [7, 8], functional relevance has been demonstrated: the ablation of cytokine receptors, such as tumor necrosis factor receptor type 1 (TNFR1) prevented memory impairment in mice fed a saturated fat-enriched diet [7]. Bioactive sphingolipids, particularly sphingosine-1-phosphate (S1P) have been increasingly recognized in the regulation of neuroinflammation [10-13]. Specifically, S1P regulates diverse cellular processes including cell survival, migration, and inflammation through its five G protein-coupled receptors (S1PR1-5) [14]. In the brain, S1P signaling influences blood-brain barrier integrity, glial activation, and neural cell communication [10, 15, 16]. S1P signaling intersects with pro-inflammatory cytokine pathways through mechanisms that include the production and release of cytokines such as TNF-α, interleukin (IL)-1β, and IL-6 [17-20] in mural cells of the cerebral vasculature, microglia, and astrocytes [19, 21, 22]. In microglia, S1P receptor signaling modulates polarization toward either pro-inflammatory or anti-inflammatory states, with S1PR1 and S1PR3 generally promoting a pro-inflammatory phenotype [23-27]. At the neurovascular interface S1P/S1PR1 signaling critically regulates adequate tight junction protein expression [28] and astrocyte function [13]. Alterations in this signaling contribute to increased blood-brain-barrier permeability [29] and glutamate-induced neurotoxicity [30], which are key events in the initiation and propagation of neuroinflammation [31] and neurodegeneration [32].

S1P is generated intracellularly by two sphingosine kinases, SphK1 and SphK2, which differ in subcellular localization, regulation, and functional outcomes [14]. While SphK1 is mainly cytosolic and often associated with pro-survival signaling [33, 34], SphK2 localizes to the nucleus and mitochondria and has been linked to both pro- and anti-inflammatory roles depending on context [35]. Intracellularly generated S1P can act independently of membrane receptors to regulate gene expression and chromatin remodeling, influencing cytokine transcription programs via both Histone Deacetylase (HDAC) inhibition [36, 37] and modulation of signal transducer and activator of transcription (STAT) signaling pathways [38]. Additionally, intracellular S1P can influence calcium homeostasis [39] and inflammasome activation [40], two processes relevant for microglial reactivity, supporting its role in shaping microglial activation independently of surface S1P-S1PR signaling. Recent studies have highlighted the relevance of SphK2 in neuroinflammatory pathways, including models of multiple sclerosis and neurodegeneration [10, 41]. However, the role of SphK2 in diet-induced neuroinflammation has not been fully elucidated. It remains hitherto unknown whether SphK2 activity contributes to microglial activation under HFD conditions, and whether these effects are region-specific within the brain.

In this study, we tested whether pharmacological SphK2 inhibition impacts microglial morphology in the cortex and hippocampus of mice exposed to HFD. These two brain regions are critically involved in cognitive processing and vulnerable to metabolic and inflammatory insults upon HFD feeding [7-9]. Our findings suggest that SphK2 mediates HFD-induced microgliosis in a brain region-specific manner, leading us to propose SphK2 inhibition as a therapeutic strategy to mitigate obesity-associated neuroinflammation.

## MATERIALS AND METHODS

### Animals

All procedures on animals were approved by the Malmö-Lund Committee for Animal Experiment Ethics (D.nr. 5.8.18-05123/2021), conducted according to EU Directive 2010/63/EU, and are reported following the ARRIVE 2.0 guidelines (Animal Research: Reporting *In Vivo* Experiments, NC3Rs initiative, UK). Male C57BL/6J mice were purchased from Taconic (Ry, Denmark) and housed at 22 °C and 50-60% humidity under a 12:12-h light-dark cycle. Food and water were provided *ad libitum*. At 9 weeks of age, mice were assigned in a cage-wise manner to either a high-fat diet (HFD; lard-base diet containing 60% kcal from fat; Research Diets, New Brunswick, NJ-USA) or a nutrient-matched control diet (CD; containing 10% kcal from fat; Research Diets, New Brunswick, NJ-USA). After 7 weeks of diet, Researcher Randomizer (randomizer.org) was used to randomly allocate mice to one of the following treatment groups (n=10/group): (i) CD + vehicle (CD; 0.3%(v/v) ethanol in 50:50 polyethylene glycol (PEG)/H_2_O); (ii) CD + SphK2 inhibitor ABC294640 (BioNordika, Solna, Sweden); CDi; 5 mg/kg in 0.3%(v/v) ethanol in 50:50 PEG/H_2_O; (iii) HFD + vehicle (HFD); or (iii) HFD + SphK2 inhibitor ABC294640 (HFDi; 5 mg/kg).

Treatments were subcutaneously (s.c.) injected every second day for two weeks. One HFDi mouse presented tumor growth at the endpoint and was therefore excluded from the study.

### Tissue Sampling

Mice under 2-3% isoflurane (IsoFlo, Abbott, Solna, Sweden) in room air were sacrificed by transcardiac perfusion with cold phosphate buffered saline (PBS; in mmol/L: 137 NaCl, 2.7 KCl, 1.5 KH_2_PO_4_, 8.1 Na_2_HPO_4_, pH 7.4) after whole blood was collected from the portal vein. Brains were collected and either fixed with 4%(w/v) paraformaldehyde (PFA) for 24 hours at 4 °C, followed by incubation in 15%(w/v) sucrose in PBS for 24 hours at 4 °C and kept in 30%(w/v) sucrose at 4 °C until further processing, or dissected before snap freezing in liquid nitrogen and kept at -80 ºC until further processing.

### Mass Spectrometry

S1P from brain tissue (for cortex, n=5/group; for hippocampus, n=5/group for CD, HFD and HFDi; n=4 for CDi) was extracted as previously described [42, 43]. Briefly, cortical and hippocampal tissue fractions were weighed, mixed with 0.5 mL PBS, and homogenized using an Ultra-Turrax TP18-10 (Janke & Kunkel KG, Germany). Homogenate volumes equivalent to 10 mg tissue were added to a glass vial, filled to 1 mL with PBS, and mixed with 100 pmol internal standard S1P-D7 (Avanti Polar Lipids, Merck, Cat#860659P) before 1 mL methanol, 200 µL hydrochloric acid (6 mol/L), and 2 mL chloroform were added to the suspension. Samples were vortexed vigorously for 3 minutes and centrifuged at 1900 x *g* for 3 minutes, whereafter the lower organic phase was transferred to a clean glass tube. Chloroform was added again, samples were vortexed and centrifuged, and the 2 organic phases were combined before chloroform was evaporated under a nitrogen stream. Dried lipids were dissolved in 200 µL methanol and transferred to auto sampler vials. A 5-point standard curve was generated in duplicates from extracts of 25 to 400 pmol S1P (Avanti Polar Lipids / Merck, Darmstadt, Germany) in 0.8%(w/v) fatty acid-free BSA/PBS (Sigma-Aldrich, Sweden, Cat#126575) applying the same extraction procedure. Samples were diluted 1:10 with methanol and analyzed by liquid chromatography-coupled tandem mass spectrometry on a 6495 QQQ instrument (Agilent Technologies, Sweden) as previously described [43].

### qPCR

Brain tissue (for cortex, n=5/group; for hippocampus, n=5/group for CD, HFD and HFDi; n=4 for CDi) was homogenized in 1x PBS using an Ultra-Turrax TP18-10 (Janke & Kunkel KG, Germany) prior to RNA isolation using TriZol Reagent (Thermo Fisher Scientific, Cat#15596018) according to manufacturer’s instructions. One µg of mRNA was reverse transcribed into cDNA using the High-Capacity cDNA Reverse Transcription Kit (Thermo Fisher, Cat#4368814) in an T100 Thermal Cycler (Bio-Rad, Hercules, CA, USA). The resulting cDNA was diluted 1:12.5 and used as template for quantitative RT-PCR in triplicates using SYBR Green PCR Master Mix (Thermo Fisher, Cat#4369702) including target-specific primers (Supplementary Table 1). Cycling and detection were carried out using C1000 Touch Thermal Cycler (Bio-Rad, Sweden, Cat#1855484). All data were normalized to the expression of 60S ribosomal protein L14 (*Rpl14*) and analyzed using a relative gene expression (*i.e.*, quantities calculated relative to the lowest Ct value).

### Immunofluorescence

Free floating 30 μm coronal brain sections (n=5/group for CD, CDi and HFD; n=4 for HFDi) were permeabilized with 0.2%(v/v) Triton X-100 in PBS for 10 min at room temperature. After PBS washing, antigen retrieval was performed using 10 mmol/L citrate buffer supplemented with 0.05%(v/v) Tween-20, for 30 min at 37 °C. Subsequently, sections were immune-stained against ionized calcium-binding adapter molecule-1 (Iba1; FUJIFILM Wako Shibayagi; Cat#019-19741, RRID: AB_839504, 1:250; or Synaptic systems; Cat#234 308, RRID:AB_2924932, 1:500) and SphK2 (Novus Cat# NBP2-52566, RRID:AB_3329346, 1:200) in a 24 well plate over night at 4 °C after blocking with blocking reagent (Roche, Sweden, Cat#11096176001) for 30 min. Sections were washed with PBS, incubated with secondary antibody (donkey anti-rabbit Alexa Fluor 594, Thermo Fisher, Cat#21207, RRID: AB_141637, 1:1,000; goat-anti guinea pig 488, Thermo Fisher, Cat#SA5-10094, RRID:AB_2556674, 1:1,000; or goat-anti mouse 647, Thermo Fisher, Cat#A21235, RRID:AB_2535804, 1:1,000) at room temperature, and mounted with Fluoromount-G mounting medium (Thermo Fisher, Cat#00-4958-02) or Fluoromount-G with DAPI (Thermo Fisher, Cat#00-4959-52). In brain slices, prefrontal cortex, *dentate gyrus* (DG) and *cornu ammonis* 1 (CA1) hippocampal regions were assessed for Iba1-positive cells (microglia) and SphK2 positivity using a Nikon A1RHD confocal microscope with a CFI Plan Apochromat Lambda 20x NA 0.75 (Nikon Instruments, Tokyo, Japan).

In cortex and hippocampal DG and CA1 regions, Iba1-positive microglia were classified into ramified (resting state; cells with a small cell body and extensive branched processes), intermediate (cells with enlarged cell body and thickened, reduced branches not longer than twice the cell body length), and amoeboid (active state; cells with round cell body without visible branches; Fig.1) [44, 45]. In addition, endpoints/cell and branch length/cell of cortical and DG microglia were quantified using the skeletonize tool in ImageJ and following a protocol previously described [46]. In brief, cells displaying their complete arborization were analyzed in max projections of 17 image stacks of 210 × 210 x 0.85 μm^3^. Total endpoints and branch length per image were calculated and an estimation of microglial arborization per cell was calculated by dividing each value by the total number of cells displayed per image.

**Figure 1.**
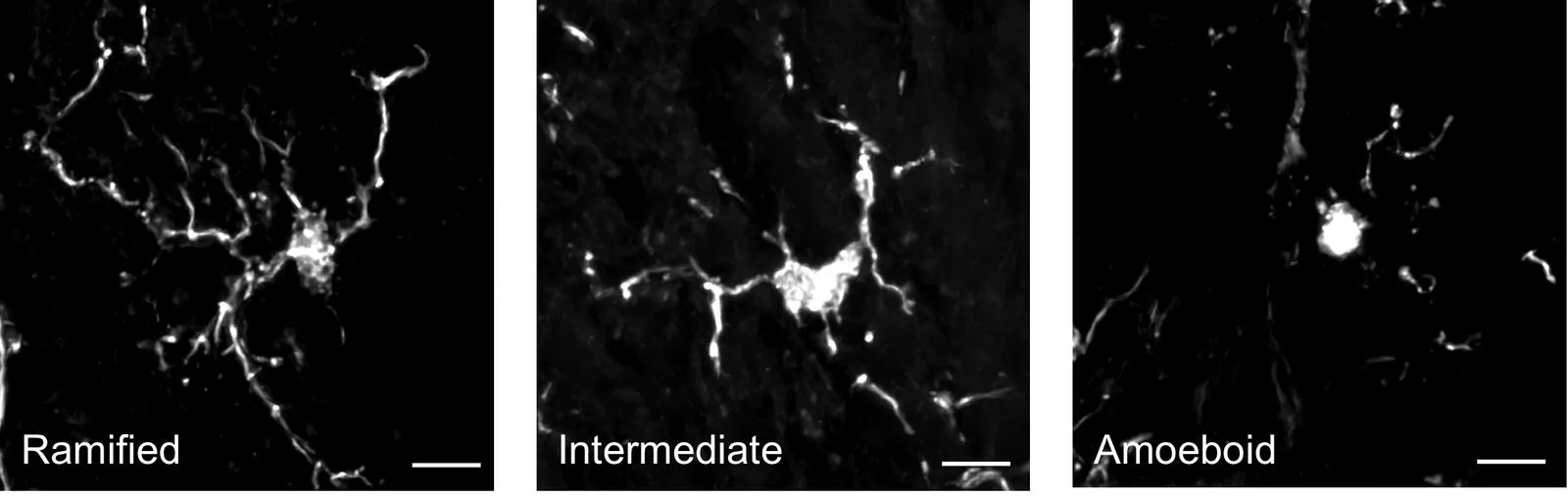
**Classification of microglial morphology**. Representative images illustrating ramified, intermediate and amoeboid microglial phenotypes. Scale bar is 10 µm.

For cortex, a total of 7 ROIs per animal were analyzed and averaged; for DG, a total of 6 to 9 images were analyzed and averaged per animal. Representative images were chosen based on median microglial activation scores and blinded morphology assessments to reduce bias.

### Western blotting

Homogenates of brain tissue fractions (for cortex, n=5/group; for hippocampus, n=5/group for CD, HFD and HFDi; n=4 for CDi) were solubilized in RIPA buffer (50 mM Tris-HCl pH 8.0, 5 mM EDTA, 1 % Triton-X, 1 % sodium-deoxycholate, 0.1 % SDS, 150 mM NaCl) supplemented with phosphatase (Abcam, Cat#ab201112) and protease inhibitors (Roche, Cat#04693124001) for 30 min on ice. Insoluble material was removed by centrifugation at 20,000 *g* at 4°C for 10 min. Protein concentration was measured using Pierce BCA Protein Assay Kit (Thermo Fisher, Cat#23227) and 30 µg of protein was mixed with 4x sample buffer (0.2 M Tris pH 6.8, 8% SDS, 40% (v/v) glycerol, 20% (v/v) β-mercaptoethanol, 0.02% (w/v) bromophenol blue) and heated for 10 min at 95°C. Proteins were loaded on 4-15 % SDS-PAGE mini gels and separated at 100 V for 2 hours. Proteins were transferred onto PVDF membranes (Bio-Rad) using a Bio-Rad Trans-Blot Turbo Transfer System (RRID:SCR_023156). Membranes were blocked for 1 h in 5 % non-fat dry milk (in phosphate-buffered saline containing 1% Tween-20 (PBST); 137 mM NaCl, 2.7 mM KCl, 10 mM Na_2_HPO_4_, 1.8 mM KH_2_PO_4_; pH 7.4) at RT and followed by incubation with primary antibody anti-phospho-S1PR1 (Thermo Fisher, Cat#PA5-40299, RRID:AB_2609210, 1:1,000) and anti-β-tubulin (Sigma-Aldrich, Cat#T4026, RRID:AB_477577, 1:5,000) overnight at 4°C. Next, membranes were incubated with HRP-conjugated goat anti-mouse (Dianova, Cat#115-035-062, 1:10,000) or goat anti-rabbit (Cell Signaling Cat#7074, RRID:AB_2099233, 1:10,000) antibody for 1 hour at RT. Proteins were visualized by enhanced chemiluminescence (SuperSignal West femto Maximum Sensitivity Substrate Thermo Fisher, Cat#34095) using a BioRad ChemiDoc MP Imaging System (RRID: SCR_019037). The developed membranes were evaluated densitometrically and relative phospho-S1PR1 protein expression normalized to β-tubulin was analyzed using Image Lab Software 6.1.0 (RRID:SCR_014210). Independent Western blot membranes were generated for each brain fraction (cortex, n = 2; hippocampus, n = 3) and subsequently normalized across blots using a common loading control that was included on every membrane.

### Statistics

Experimenters were blinded to grouping during sample analysis and data processing, using identification codes that concealed the identity of the intervention. Results were analyzed using GraphPad Prism 9 (San Diego, California) and MATLAB (MathWorks, Natick, MA-USA). The sample size (N) refers to the number of animals.

All data sets with N<6 are presented as median ± interquartile range and were analyzed using non-parametric methods to account for small group sizes. Specifically, we used the aligned rank transform (ART) ANOVA for grouped (factorial) analyses and the Mann Whitney test for comparisons of two groups. Significance was defined as p < 0.05. Statistical testing results are detailed in Supplementary Table 2.

In addition, for datasets that appeared normally distributed based on Q-Q plot inspection, we report results from parametric testing using t-tests or standard ANOVAs followed by post hoc analysis with Fisher’s least significant difference (LSD) test (Supplementary Table 3).

## RESULTS

### Region-Specific Expression of Sphk2 Suggests a Potential Role in HFD-Induced Brain Changes

To identify brain regions with potential susceptibility to SphK2-mediated signaling, we first profiled the regional mRNA expression of *Sphk1* and *Sphk2* in cortex and hippocampus. Under CD conditions, *Sphk2* trended towards a more abundant expression than *Sphk1* in both brain regions but did not reach significance (Fig. 2A). Importantly, neither *Sphk1* nor *Sphk2* mRNA levels were altered by HFD exposure in either region (Fig. 2B-C).

**Figure 2.**
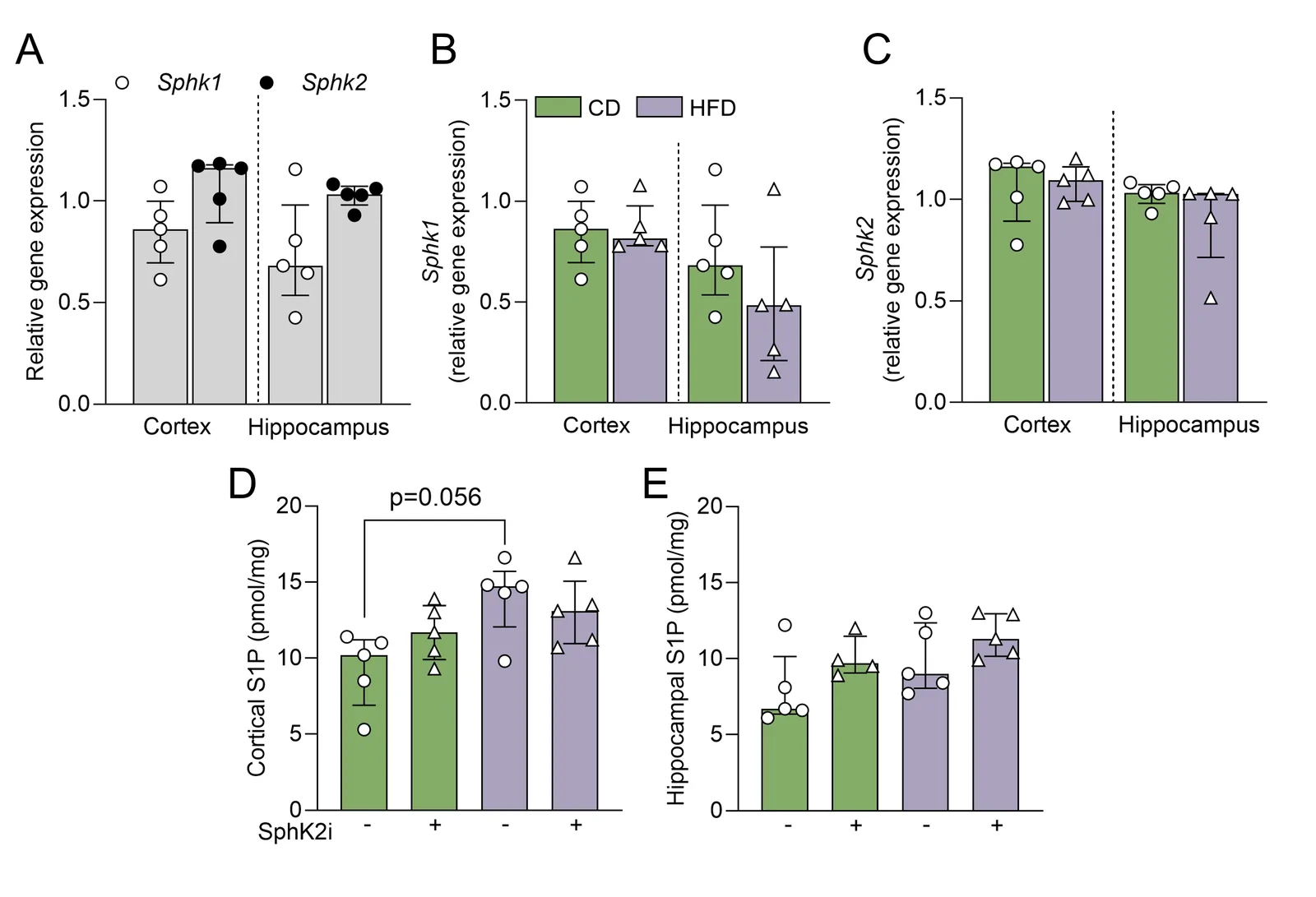
**Differential expressions of S1P-generating enzymes and regional S1P levels in the mouse brain under CD and HFD feeding. (A)** *Sphk1 and Sphk2* mRNA expression levels in cortex and hippocampus of mice fed a CD for 9 weeks. Differences of (B) *Sphk1* and (C) *Sphk2* expression levels between CD and HFD-fed mice at 9 weeks of diet exposure. (**D**) S1P levels in cortex of CD, CDi, HFD and HFDi mice. (**E**) S1P levels in hippocampus of CD, CDi, HFD and HFDi mice. For panel, A-D, n=5/group; for panel E, n=5/group for CD, HFD and HFDi and n=4 for CDi; data are presented as median ± IQR. Panel (A) is compared with Mann-Whitney tests. Panels (B-C) are compared with unpaired t-tests. Panels (D-E) are compared with a 2-way ART-ANOVA followed by Mann-Whitney tests for multiple comparisons. *CD - control diet, HFD - high-fat diet, S1P - sphingosine-1-phosphate, Sphk - sphingosine kinase, SphK2i - sphingosine kinase 2 inhibitor.*

We next examined tissue S1P levels as a functional readout of sphingosine kinase activity. Baseline S1P levels were not different between the cortex and the hippocampus (CD, ctx = 9.28 pmol/mg, CD, hippo = 7.94 pmol/mg; p = 0.420). In comparison to CD, HFD feeding augmented S1P levels in the cortex (p = 0.056; Fig. 2D, diet effect: p = 0.019) to a larger extent than in the hippocampus (p = 0.151; Fig. 2E, diet effect: p = 0.026). These findings indicate that cortical S1P accumulation may be particularly responsive to HFD and suggest differential regulation of sphingolipid metabolism across brain regions. This interpretation is supported by regionally divergent expression of S1P signaling components: consistent with the region-specific increase in S1P levels, HFD induced a significant downregulation of *S1pr1* expression in the cortex (p = 0.032, Fig. 3B; diet effect: p = 0.037), but not in the hippocampus (p = 0.814, Fig. 3E). This was accompanied by increased levels of phosphorylated S1PR1 in the cortex (p = 0.032, Suppl. Fig. 1A, diet effect: p = 0.024) but not the hippocampus of HFD mice (diet effect: p = 0.814, Supplementary Fig. 1B), suggesting receptor internalization in response to S1P elevation [47]. HFD did not alter the expression of *S1pr*2 or *S1pr3* in either brain region (Supplementary Fig. 2). Further diet effects were observed for *Sgpp1* expression in the hippocampus (diet effect: p = 0.013, Fig. 3D) but not the cortex (diet effect: p = 0.114, Fig. 3A), while *Sgpp2* and *Sgpl1* expression were not altered under HFD conditions (Fig. 3C & 3F, Supplementary Fig. 2C & 2F). Treatment with the SphK2 inhibitor did not significantly alter S1P concentrations in either brain region (Fig. 2D-E), although hippocampal S1P levels were highest in the HFDi group (Fig. 2E). Notably, *Sgpp1* expression was significantly reduced in the hippocampus of HFDi mice (p = 0.032, Fig. 3D, diet effect: p = 0.013), while *Sgpp2* expression was decreased in the cortex with no effects on S1P levels (p = 0.032, Fig. 3C, interaction: p = 0.005). No changes were observed in *Sgpl1* expression following SphK2i treatment in either brain region (Supplementary Fig. 2C & 2F), nor in hippocampal *Sgpp2* expression (p = 0.095, Fig. 3F, treatment effect: p = 0.029). Notably, *Sphk1* and *Sphk2* expression were largely unaltered by SphK2i treatment in both cortex and hippocampus (Supplementary Fig. 3A-D).

**Figure 3.**
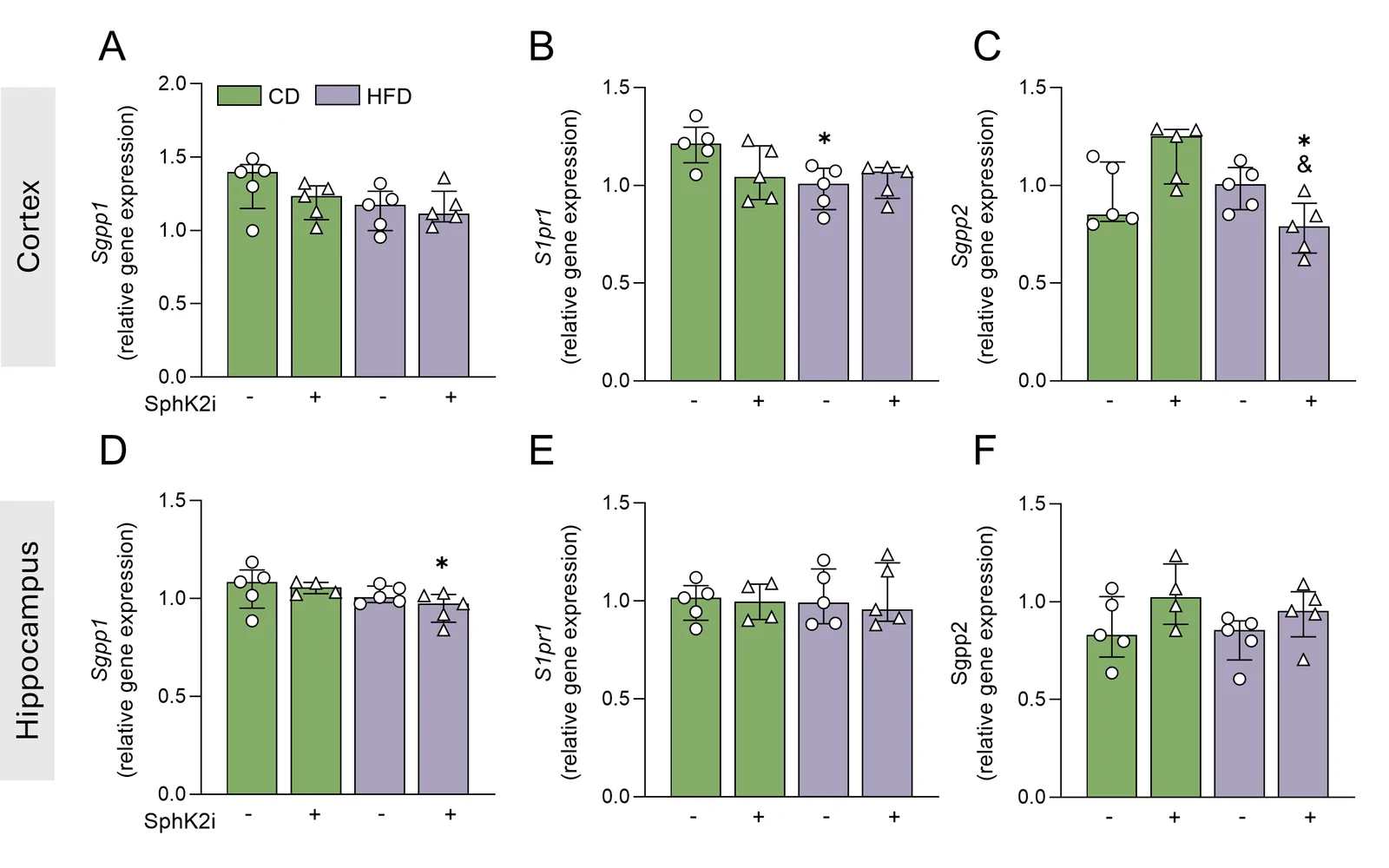
**S1P phosphatases and *S1pr1* expression are differently affected in cortex and DG**. Expression of (A) *Sgpp1*, (B) *S1pr1* and (C) *Sgpp2* in the cortex of CD, CDi, HFD and HFDi mice. Expression of (D) *Sgpp1*, (E) *S1pr1* and (F) *Sgpp2* in the hippocampus of CD, CDi, HFD and HFDi mice. For panel, A-C, n=5/group; for panel D-F, n=5/group for CD, HFD and HFDi and n=4 for CDi; data are presented as median ± IQR. Data are compared with a 2-way ART-ANOVA followed by Mann-Whitney tests for multiple comparisons. * p < 0.05 for comparisons to corresponding CD group; & p < 0.05 for comparisons to corresponding vehicle group. CD - control diet, HFD - high-fat diet, *S1pr* - sphingosine-1-phosphate receptor, *Sgpp* - sphingosine phosphatase, SphK2i - sphingosine kinase 2 inhibitor.

### SphK2 Inhibition Ameliorates HFD-associated Microglial Activation in Cortex and Hippocampal Dentate Gyrus

SphK2i-treatment resulted in exacerbated weight gain in the HFD group but not the CD group (Supplementary Fig. 4, interaction: p = 0.003, diet effect: p < 0.0001), indicating that the inhibitor may affect systemic metabolic responses under obesogenic conditions. Given the differential S1P distribution and expression of *Sphk2*, we next determined microglial morphology to assess their activation status as a marker of neuroinflammation [48]. HFD feeding induced pronounced microglial activation in the cortex and both hippocampus regions (DG and CA1), evident from reduced ramification and a transition to a more amoeboid morphology (Fig. 4A-C, morphology x diet: p < 0.0001). In contrast, mRNA expression of canonical cytokine markers (IL-6, TNF-α, IL-1β) in cortex and hippocampus remained unchanged by HFD (Supplementary Table 4), suggesting that morphological microglial activation precedes or occurs independently of overt cytokine induction. Moreover, expression levels of *Cd68* and *Nos2* remained unaffected by HFD (Supplementary Table 4). Strikingly, treatment with SphK2i abolished the HFD-induced morphological changes in microglia only in the DG of the hippocampus evidenced by significantly higher proportions of ramified microglia (p = 0.016) and significantly lower proportions of amoeboid microglia (p = 0.032, Fig. 4B), implicating a role for SphK2 in HFD-induced reactive microgliosis. Microglial cell numbers remained unaltered across HFD groups, suggesting that SphK2 inhibition primarily affects activation state rather than cell proliferation under HFD conditions (Fig. 4D-F). In CD, however, SphK2i lowered the proportion of amoeboid microglia in the cortex and (p = 0.008, Fig. 4A) and reduced the number of microglia in the DG (p = 0.048, Fig. 4E, treatment effect: p = 0.037).

**Figure 4.**
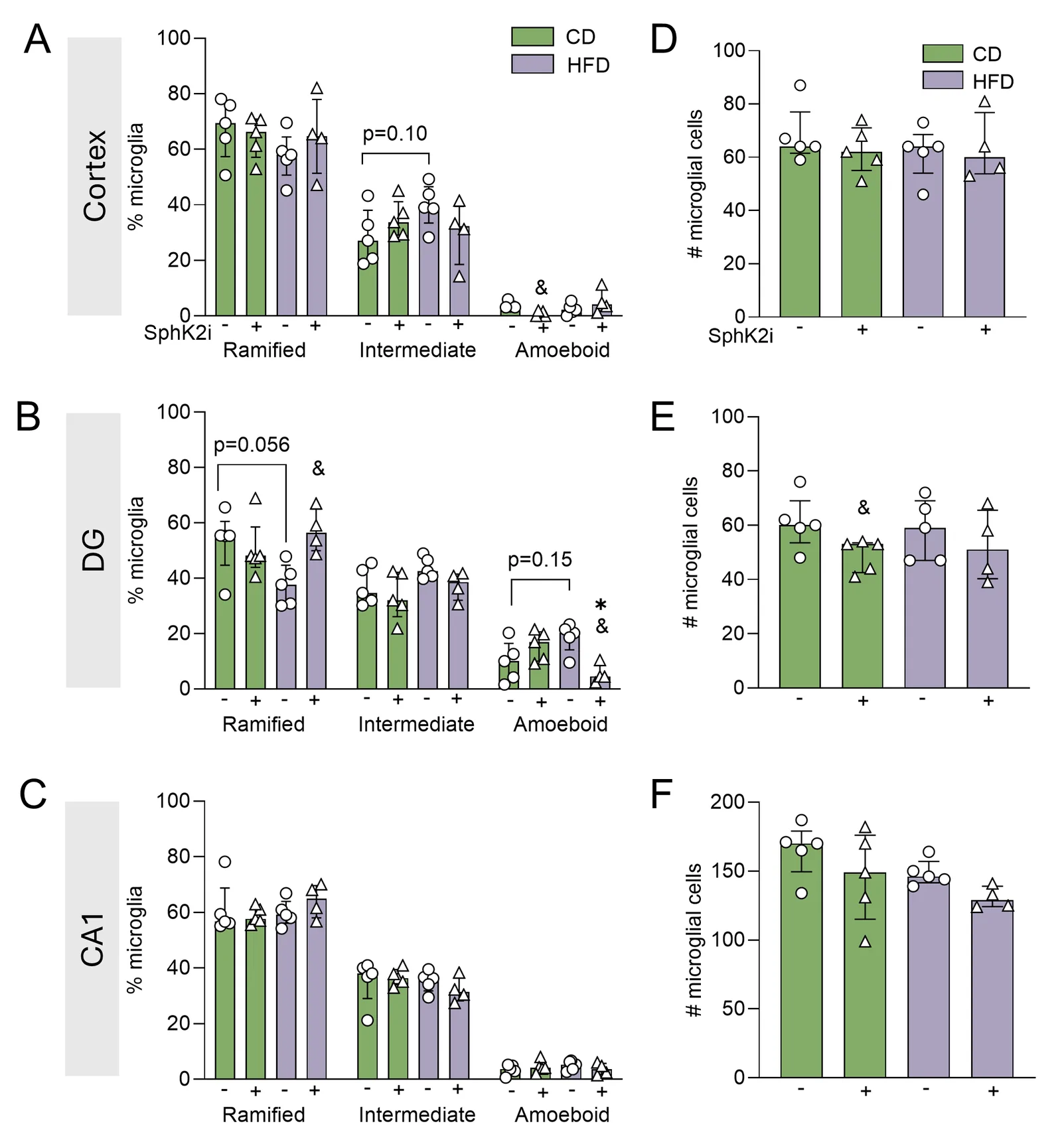
**SphK2 inhibition attenuates microglial activation in the cortex and DG following HFD**. Iba1-positive microglia were classified as ramified, intermediate, or amoeboid based on their morphology in (A) cortex as well as (B) DG and (C) CA1 hippocampal regions. (**D-F**) Quantification of microglial cells in the three regions; cortex, DG and CA1. Analysis from n= 6-10 ROI per brain area in n=4-5 mice per group (n=5 for CD, CDi and HFD; n=4 for HFDi). Panels are presented as median ± IQR. Panels (A-C) are compared with a 3-way ART-ANOVA followed by Mann-Whitney tests for multiple comparisons. Panels (D-F) are compared with a 2-way ART-ANOVA followed by Mann-Whitney tests for multiple comparisons. * p < 0.05 for comparisons to corresponding CD group; ^&^ p < 0.05 for comparisons to corresponding vehicle group. *CA1 - cornu ammonis 1, CD - control diet, DG - dentate gyrus, HFD - high-fat diet, Iba1 - Ionized calcium-binding adaptor molecule 1, SphK2i - sphingosine kinase 2 inhibitor.*

To further quantify these changes, microglial endpoints and total branch length per cell in branched cells was analyzed using a semi-automated approach [46]. HFD-fed mice displayed significantly reduced number of endpoints and lower branch length per cell in both cortex and DG compared to CD-fed controls, confirming microglial activation (Fig. 5). SphK2i treatment improved microglial complexity in the cortex, evidenced by a similar-to-control number of endpoints per cell (Fig. 5A-B, treatment effect: p = 0.172), while the rescue in branch length per cell was incomplete (Fig.5C, treatment effect: p = 0.058). In contrast, affected endpoints and branch length per cell were not affected by SphK2i treatment in the DG region (Fig. 5D-F, treatment effect: p = 0.638), suggesting a region-specific efficacy of SphK2 inhibition. Qualitative assessment of SphK2 protein localization revealed distinct, brain region-specific patterns in microglia. In the cortex, SphK2 immunoreactivity was largely absent from Iba1-positive microglia (Supplementary Fig. 5). In contrast, the hippocampal CA1 region, and to a lesser extent the dentate gyrus, displayed SphK2 staining in microglia soma (Supplementary Fig. 5). These findings indicate that the impact of SphK2 on microglial morphology under HFD conditions may be determined by both its region-specific expression and its subcellular compartmentalization.

**Figure 5.**
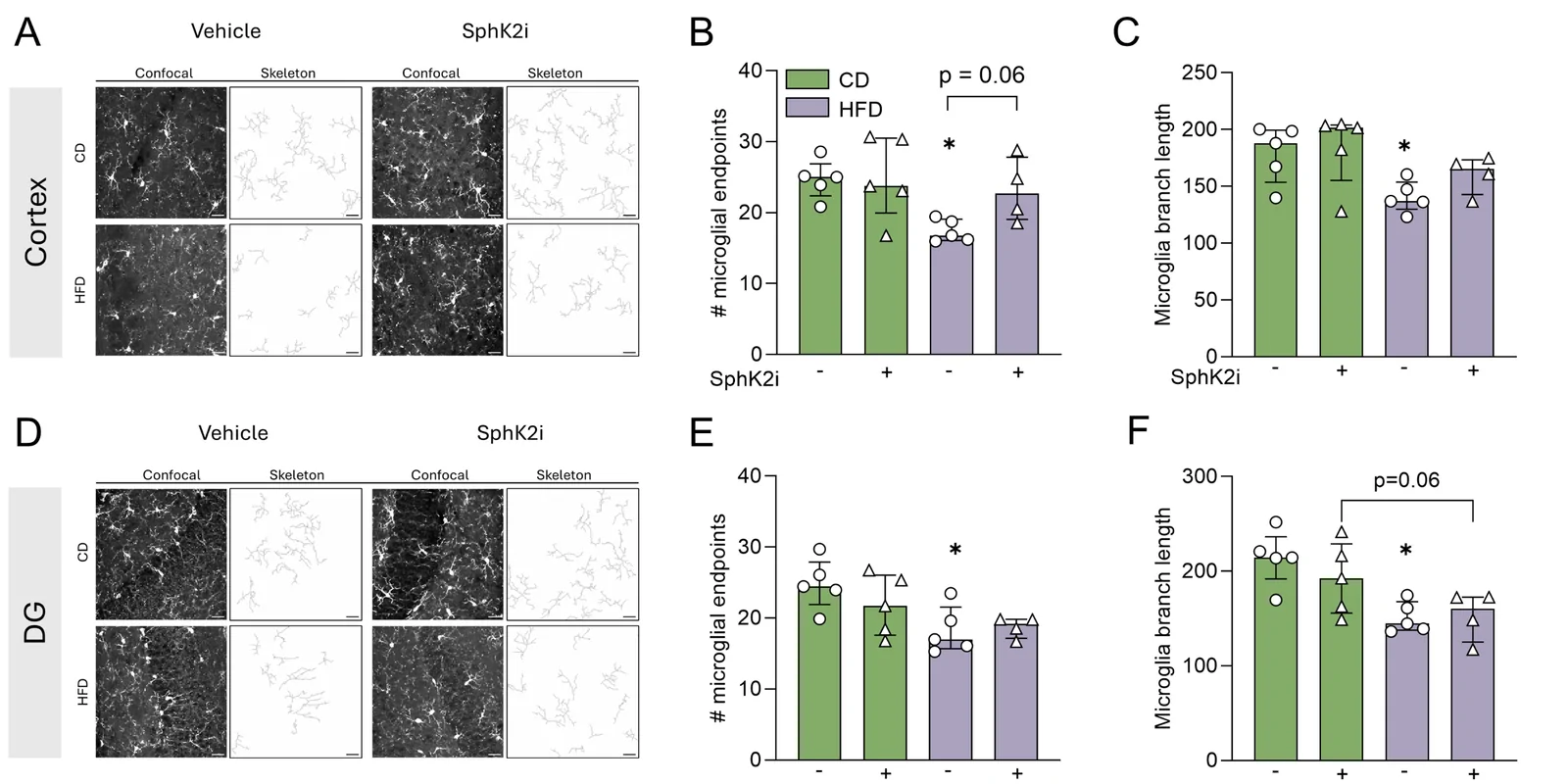
**SphK2 inhibition attenuates the HFD-induced retraction of microglial processes in the cortex and DG**. (**A**) Representative images of Iba1-positive cortical microglia and skeletons utilized for assessment of number of endpoints and branch length of CD, CDi, HFD and HFDi mice. Scale bars 20 μm. (**B**) Quantification of number of endpoints/cell in cortical microglia of CD, CDi, HFD and HFDi mice. (**C**) Quantification of average branch length/cell of cortical microglia of CD, CDi, HFD and HFDi mice. (**D**) Representative images of Iba1-positive DG hippocampal microglia and skeletons utilized for number endpoints and branch length of CD, CDi, HFD and HFDi mice. Scale bars 20 μm. (**E**) Quantification of number of endpoints/cell in DG microglia of CD, CDi, HFD and HFDi mice. (**F**) Quantification of average branch length/cell of DG microglia of CD, CDi, HFD and HFDi mice. Analysis from n= 6-7 ROI per brain area in n=4-5 mice per group (n=5 for CD, CDi and HFD; n=4 for HFDi). Data are presented as median ± IQR and are compared with a 2-way ART-ANOVA followed by Mann-Whitney tests for multiple comparisons. * p < 0.05 for comparisons to corresponding CD group. *CD - control diet, DG - dentate gyrus, HFD - high-fat diet, Iba1 - Ionized calcium-binding adaptor molecule 1, SphK2i - sphingosine kinase 2 inhibitor.*

## DISCUSSION

This study identifies a brain region-specific role for SphK2-S1P signaling in mediating neuroinflammatory responses to HFD feeding. We show that cortical but not hippocampal S1P levels increase in response to HFD. HFD-associated S1PR1 internalization in the cortex without apparent microglial SphK2 expression suggests a receptor-driven, extracellular S1P-S1PR1 signaling axis, likely involving non-microglial S1P sources. In contrast, hippocampal microglia express SphK2 but lack S1PR1 internalization in response to HFD feeding, supporting a primarily intracellular S1P signaling mode, potentially regulating microglial morphology and activation via non-canonical, receptor-independent pathways (Fig. 6). Notably, pharmacological inhibition of SphK2 effectively attenuated HFD-induced microglial activation in both the cortex and the DG region of the hippocampus, with partial restoration of microglial arborization in the cortex, suggesting that SphK2 contributes to the propagation of HFD-induced neuroinflammatory signals in a region-dependent manner.

**Figure 6.**
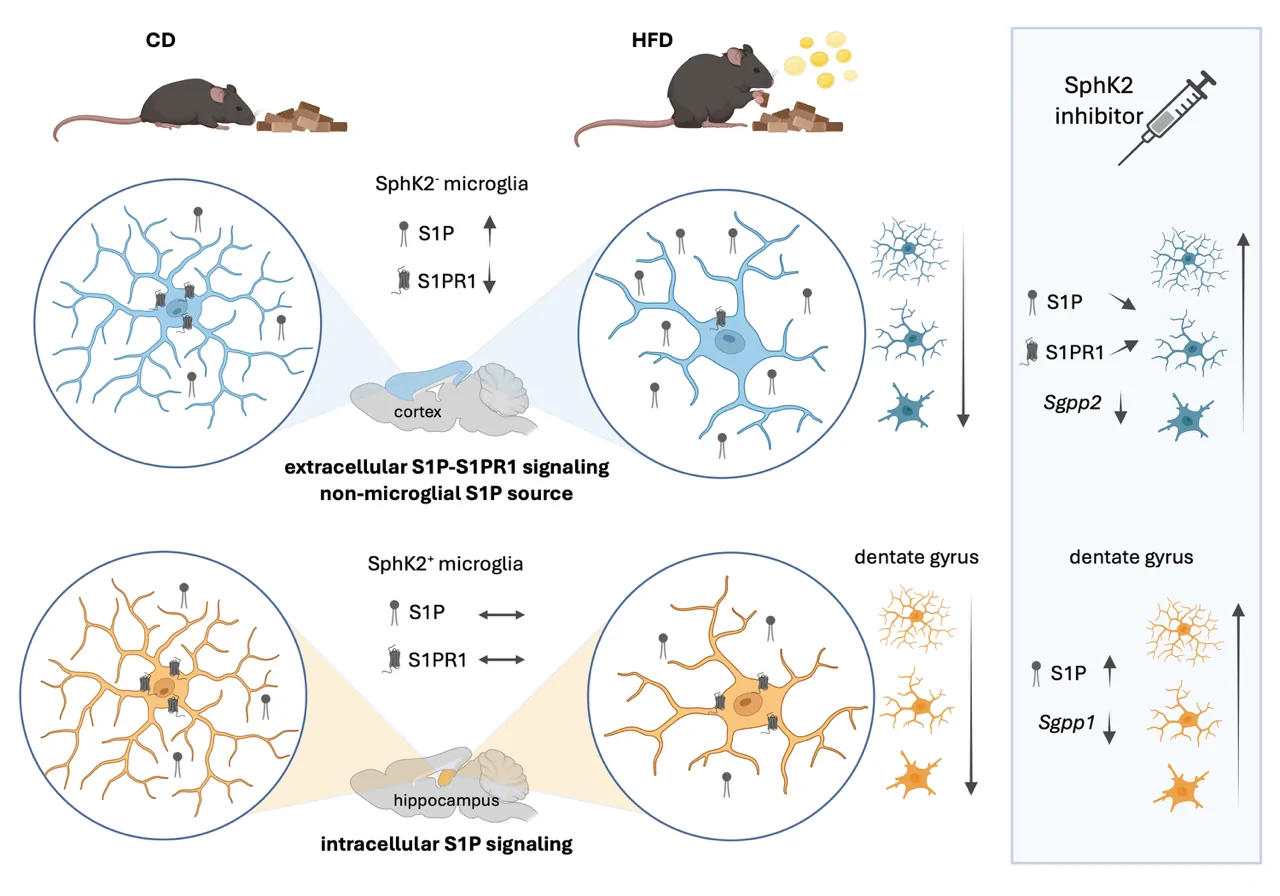
**Brain region-specific compartmentalization of SphK2-S1P signaling involved in microgliosis**. High-fat diet (HFD) may induce microglial activation through distinct sphingosine-1-phosphate (S1P) signaling mechanisms in cortex and hippocampus. In the cortex, S1P receptor 1 (S1PR1) downregulation and increased phosphorylated S1PR (pS1PR1) immunoreactivity indicate receptor activation and internalization, suggesting a predominant extracellular S1P-S1PR1 signaling axis originating from non-microglial cells as microglial sphingosine kinase 2 (SphK2) expression is neglectable in this region. In contrast, in the hippocampus, SphK2 expression co-localizes with Iba1-positive microglia without S1PR1 expression alterations or receptor internalization, indicating a primarily intracellular, receptor independent S1P signaling mode. This regional compartmentalization of SphK2-S1P signaling may underlie divergent mechanisms of HFD-induced microgliosis and differential sensitivity to SphK2 inhibition. CD - control diet, HFD - high fat diet, S1P -sphingosine-1-phosphate, S1PR1 - sphingosine-1-phosphate receptor 1, SphK2 - sphingosine kinase 2, Sgpp - sphingosine phosphatase. Image created with BioRender.

The observed accumulation of S1P in the cortex following HFD aligns with previous studies showing that metabolic stress enhances sphingolipid signaling in the CNS [49]. This elevation is paralleled by downregulation of *S1pr1* expression and concomitant receptor phosphorylation in the cortex but not the hippocampus, suggesting receptor desensitization and internalization as negative feedback in response to ligand accumulation. Since internalization of S1PR1 is a well-recognized consequence of ligand-induced receptor activation, these findings imply that extracellular S1P release in the cortex is sufficient to trigger canonical S1PR1 signaling. The absence of changes in *S1pr2/3* expression in either region suggests that S1PR1 is the predominant receptor subtype engaged under HFD conditions in the cortex. Elevated cortical S1P levels may reflect increased SphK2 activity or reduced S1P degradation in this region. Although expression of *Sphk2* or most of the other S1P degrading enzymes was not affected by HFD, their enzymatic activity could still be influenced by metabolic cues (palmitate is abundant in HFD [7], and is activated to palmitoyl-CoA that drives ceramide and sphingosine synthesis) or post-translational modifications [50]. The fact that SphK2 inhibition reduced microglial activation without altering *Sphk2* transcription suggests that SphK2 activity, rather than expression, is the critical factor modulating microglial phenotype under HFD conditions. Surprisingly, we have not observed a reduction of S1P concentrations in cortex and hippocampus upon SphK2 inhibition. In contrast, the highest hippocampal S1P levels were observed in the HFDi group and were paralleled by an HFD-associated lowering of hippocampal *Sgpp1* expression. Hence, it should be acknowledged that (i) there might be compensatory enzyme activity changes by SphK1 or S1P degrading enzymes, and (ii) cellular-specific S1P changes might not be reflected by the analysis of whole tissue from different brain regions. In fact, lipid profile heterogeneity within the brain is known, and has been supported by single cell lipidomic studies [51, 52]. Noteworthy, proteomics in cultured cells has revealed higher SphK2 density in microglia than neurons or astrocytes [53], which further advocates for a specific role of SphK2 inhibition in the control of reactive microgliosis.

In this context, it is also important to consider that SphK1 contributes substantially to the circulating S1P pool [54-56], which is essential for vascular and barrier integrity [57]. Therefore, while dual SphK1/SphK2 inhibition may represent a strategy to lower brain S1P more broadly, such approaches may also reduce plasma S1P levels and thereby impair endothelial S1PR1 signaling with possible detrimental effects on blood-brain barrier function [58]. Future studies with careful dosing and compartment-specific targeting will be needed to test whether combined inhibition can effectively modulate neuroinflammation without compromising vascular homeostasis.

Our findings are consistent with prior evidence implicating S1P signaling in microglial activation [59, 60], including evidence showing that S1P promotes microglial motility via crosstalk with purinergic signaling pathways [61]. Moreover, S1P has been shown to enhance pro-inflammatory cytokine production (*e.g.*, TNF-α, IL-1β), induce iNOS elevation [62, 63], and promote microglial chemotaxis *in vitro* [61], while S1PR antagonism can dampen neuroinflammatory responses *in vivo* [64]. The mechanistic crosstalk between sphingolipids and cytokine signaling remains insufficiently explored in the brain, despite growing evidence from cardiovascular and cancer models [65-69]. Earlier work has shown that TNF-α can activate SphK1 in cerebral artery smooth muscle, thereby augmenting cerebrovascular tone with consequences for brain blood flow [70, 71]. More specifically, microglia activation and alterations of neuronal arborization and spine density were associated with augmented TNF-α signaling [72]. Lastly, TNF-α was shown to reduce the expression of the cystic fibrosis transmembrane conductance regulator (CFTR), thereby limiting intracellular S1P degradation [73]. Since CFTR is also expressed on microglia and neurons [44], this regulatory mechanism could influence intra- and extracellular S1P concentrations and signaling under neuroinflammatory conditions. These interactions merit further investigation in the context of metabolic and neuroinflammatory disease.

Thus far, most studies investigating S1P signaling and neuroinflammation have focused on targeting S1PR signaling and SphK1 [64, 74, 75]. Here, we provide evidence for a selective role of SphK2 in HFD-induced microgliosis, extending prior findings and highlighting the therapeutic potential of isoform-specific targeting. Interestingly, SphK2 inhibition rescued microglial morphology more robustly in the cortex than in the DG, suggesting region-specific differences in microglial sensitivity to S1P and/or in SphK2-dependent signaling pathways. Microglial heterogeneity across brain regions is increasingly recognized, with differences in transcriptional profiles, basal activation states, and responsiveness to stimuli [76]. It is therefore plausible that cortical microglia are more dependent on SphK2-derived S1P signaling than hippocampal microglia, or that compensatory pathways in the DG blunt the effects of SphK2 inhibition. Another important factor may be regional differences in S1PR subtype expression [49, 77]. Since S1P signals through five distinct S1PRs, each engaging specific downstream pathways, local receptor profiles likely shape microglial responses. For example, S1PR2 has been linked to S1P-induced microglial production of IL-6 [78] and the S1PR1 is implicated as a regulator of cytokine mRNA expression in LPS-stimulated microglia [27]. Interestingly, gene expression profiling supports differential and brain region-specific alterations of *S1pr1* in response to HFD. If cortical microglia rely more heavily on S1PR1-driven pro-inflammatory signaling [64, 79, 80], SphK2 inhibition may exert stronger anti-inflammatory effects in this region, whereas hippocampal microglia expressing other S1PR subtypes (*e.g.*, S1PR2, S1PR3 [81]) or lower S1PR levels may be less sensitive to changes in S1P levels [27]. Such S1PR-specific differences could contribute to the regionally distinct microglial responses we observed following SphK2 inhibition. Future studies characterizing S1PR expression across brain regions under HFD conditions will be essential to clarify these mechanisms.

Importantly, HFD exposure is known to induce microgliosis with negligible effects on microglia proliferation in the mouse cortex and hippocampus [8]. Similarly, microglial cells exposed to the saturated fatty acid palmitate show negligible proliferation when compared to traditional infectious stimuli [82]. Our results demonstrate that the number of microglia also remains unchanged upon SphK2 inhibition und HFD conditions, indicating that SphK2 modulates microglial activation rather than proliferation or survival. This distinction reinforces the view that SphK2 acts as a functional regulator of microglial state, possibly via intracellular signaling or epigenetic modulation, rather than by altering cell population dynamics. Beyond its enzymatic role in generating S1P, SphK2 has also been reported to function as a transcriptional regulator by localizing to the nucleus and interacting with histone deacetylases, influencing gene expression independently of its kinase activity [37, 83]. This non-canonical nuclear function may further contribute to SphK2’s role in shaping microglial activation and inflammatory gene expression [84].

These findings have broader implications for understanding the molecular links between obesity, neuroinflammation, and cognitive decline. SphK2 may serve as a metabolic sensor that couples lipid excess to neuroimmune activation, and its inhibition could represent a strategy to prevent or reverse CNS complications of obesity. In the current study, behavioral assays were not performed and should be included in future *in vivo* work to study the effect of SphK2 inhibition on the relation between microglial activation and cognitive impairment. Previous studies have demonstrated the involvement of neuroinflammation and microglial alterations on memory performance in HFD -induced obesity and other models [85-87]. Future studies should explore the downstream effectors of SphK2 activity in microglia, including S1PR-dependent and -independent pathways, and assess the impact of chronic SphK2 inhibition on behavior and cognition in HFD-fed animals.

SphK2 inhibition has recently been proposed to prevent weight gain during HFD feeding, while having no therapeutic effect in models of obesity and metabolic syndrome [88]. In our study, SphK2 inhibition exacerbated weight gain in HFD-fed mice without significantly impacting weight gain of control mice. This suggests that the observed beneficial effects of inhibiting SphK2 are independent of systemic actions that mitigate obesity.

In summary, this study reveals that SphK2 contributes to HFD-induced microglial activation in a region-specific manner, with selective SphK2 inhibition mitigating cortical and, to a lesser extent, hippocampal reactive microgliosis. Importantly, SphK2 is differentially expressed across brain regions and cell types. In the cortex, SphK2 was not detectable in Iba1-positive microglia, whereas in the DG and CA1 hippocampal regions, SphK2 was expressed in microglia, with mainly cytoplasmic localization. This spatial segregation raises the possibility that the functional consequences of SphK2 activation differ between brain regions. While in the cortex, extracellular S1P may be produced predominantly by non-microglial cells, leading to paracrine activation and internalization of S1PR1 on target cells, hippocampal SphK2 is positioned within microglia themselves. Here, cytosolic SphK2 may locally modulate cytoskeleton-dependent morphological changes, while nuclear SphK2 could contribute to gene regulation via intracellular S1P-mediated inhibition of histone deacetylases independent of canonical S1PR1 activation (Fig. 6). This brain region-specific divergence highlights the need to consider both the cellular source of S1P and the subcellular localization of SphK2 when interpreting the functional impact of S1P pathway modulation in the context of metabolic and neuroinflammatory disorders.

## Supplementary Materials

The Supplementary data can be found online at: www.aginganddisease.org/EN/10.14336/AD.2025.0636.
