# Supplementary material for: Sphingosine Kinase 2 Inhibition Ameliorates Neuroinflammation in Diet-Induced Obese Mice
Source: Aging Dis. 2026 Sep 3;17(6):3305–19. doi: 10.14336/AD.2025.0636 (PMC13653697; doi:10.14336/AD.2025.0636)
Supplement: Supplementary file 1 [file AD-17-6-3305-s.pdf]

SUPPLEMENTARY DATA

Sphingosine Kinase 2 Inhibition Ameliorates Neuroinflammation in Diet-Induced Obese Mice

Lotte Vanherle, Cecilia Skoug, Lisa Teresa Porschen, Fátima Gimeno-Ferrer, Frank Matthes, João M. N. Duarte, Anja Meissner

© 2025. Vanherle L et al. Published online at http://www.aginganddisease.org/EN/10.14336/AD.2025.0636

SUPPLEMENTARY DATA

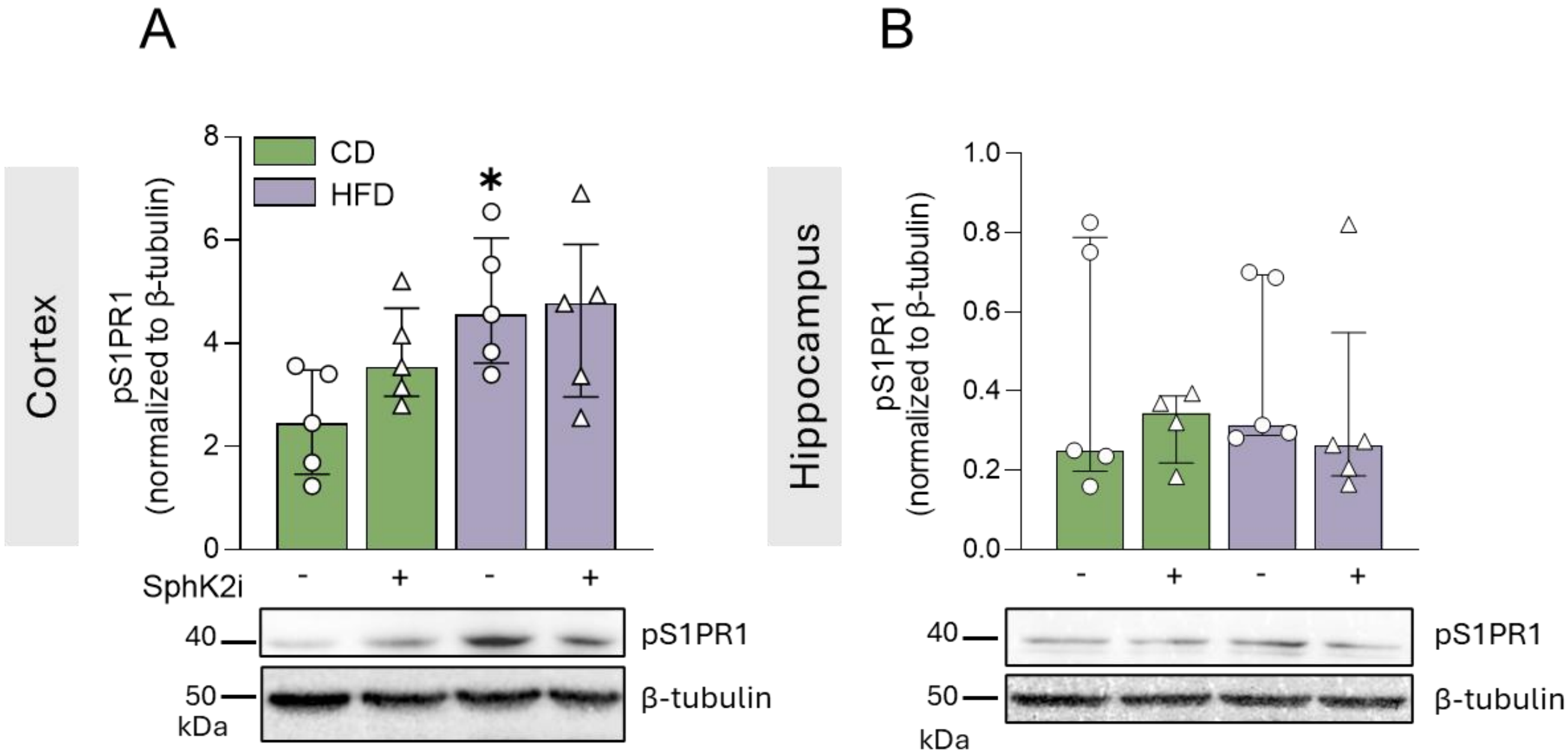

Supplementary Figure 1. High-fat diet (HFD) increases phosphorylated sphingosine-1-phosphate receptor 1 (pS1PR1) expression in cortex but not hippocampus. Protein expression of pS1PR1 **(A)** is increased in the cortex by HFD feeding but **(B)** remains unchanged in the hippocampus. For panel A: n = 5 per group. For panel B: n = 5 per group for control diet (CD), HFD, and high-fat diet plus sphingosine kinase 2 inhibitor (HFDi); n = 4 for control diet plus sphingosine kinase 2 inhibitor (CDi). Data are presented as median ± interquartile range (IQR) and analyzed by two-way ART-ANOVA followed by Mann-Whitney multiple comparisons. *p < 0.05 vs. corresponding CD group. *SphK2i - sphingosine kinase 2 inhibitor, pS1PR1 – phosphorylated sphingosine-1-phosphate receptor 1.*

© 2025. Vanherle L et al. Published online at http://www.aginganddisease.org/EN/10.14336/AD.2025.0636

SUPPLEMENTARY DATA

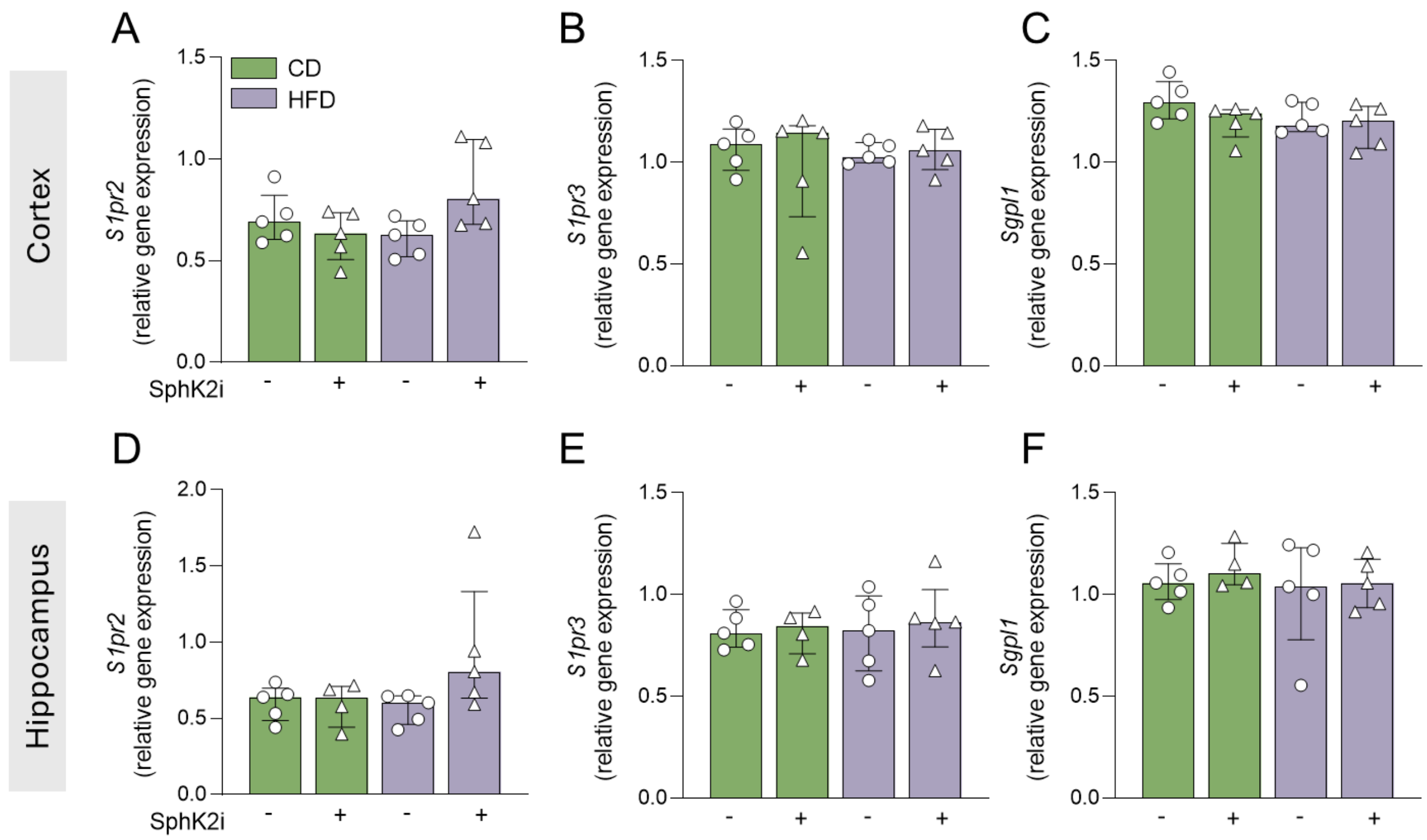

Supplementary Figure 2. S1pr2, S1pr3, and Sgpl1 expression are unaffected in cortex and hippocampus. Expression of **(A)** sphingosine-1-phosphate receptor 2 (*S1pr2*), **(B)** sphingosine-1-phosphate receptor 3 (*S1pr3*), and **(C)** sphingosine lyase (*Sgpl1*) in the cortex of mice fed a control diet (CD), control diet plus sphingosine kinase 2 inhibitor (CDi), high-fat diet (HFD), or high-fat diet plus sphingosine kinase 2 inhibitor (HFDi). Panels D-F show expression of the same genes in the hippocampal DG of the same groups. For panels A-C: n = 5 per group. For panels D-F: n = 5 per group for CD, HFD, and HFDi; n = 4 for CDi. Data are presented as median ± interquartile range (IQR) and analyzed by two-way ART-ANOVA followed by Mann-Whitney multiple comparisons. *SphK2i - sphingosine kinase 2 inhibitor, S1pr2 - sphingosine-1-phosphate receptor 2, S1pr3 - sphingosine-1-phosphate receptor 3, Sgpl1 - sphingosine lyase.*

© 2025. Vanherle L et al. Published online at http://www.aginganddisease.org/EN/10.14336/AD.2025.0636

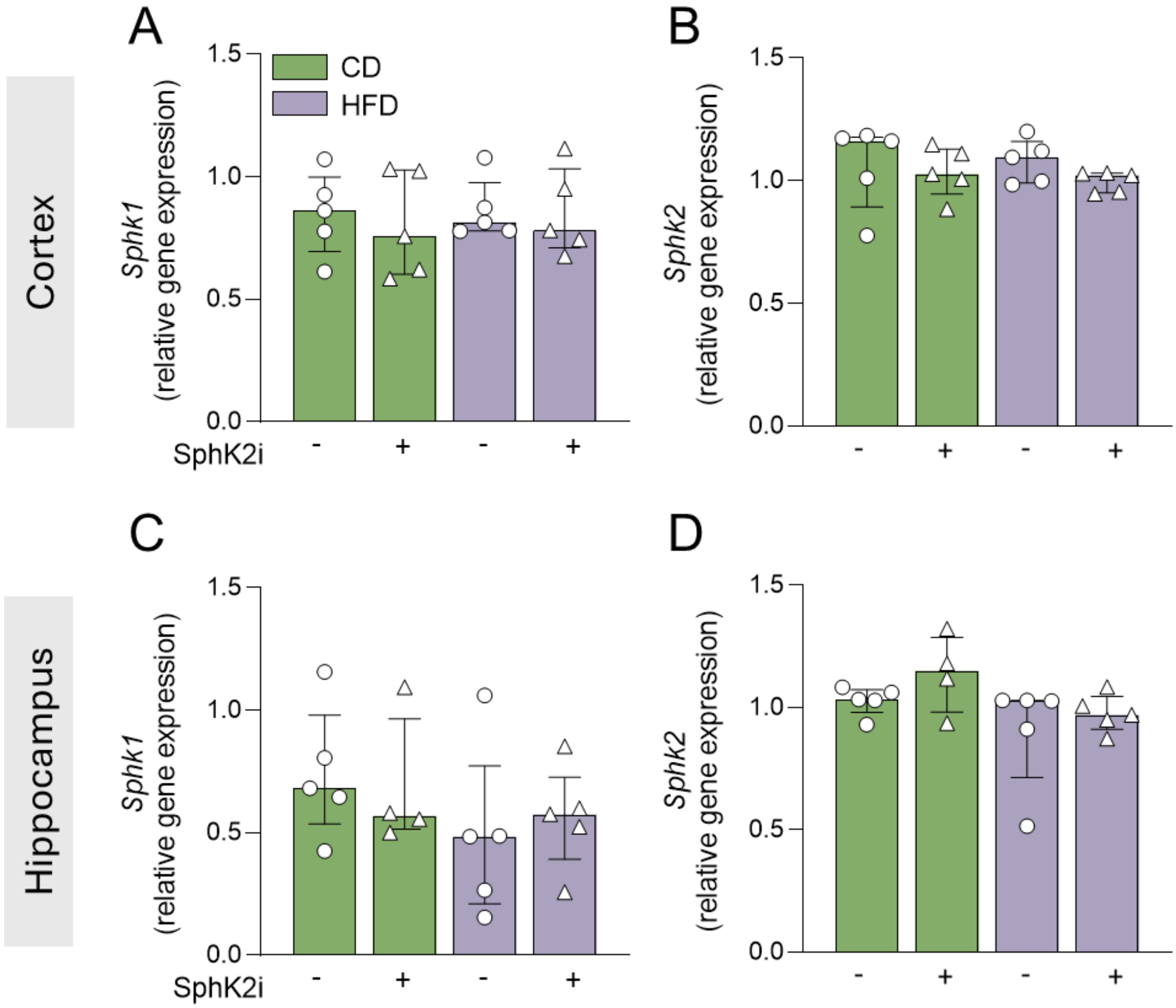

Supplementary Figure 3. *Sphk1* and *Sphk2* expression are unaffected by HFD or SphK2 inhibition in cortex and hippocampus. Expression of **(A)** *Sphk1* and **(B)** *Sphk2* in the cortex of mice maintained on control diet (CD), control diet with SphK2 inhibitor (CDi), high-fat diet (HFD), or high-fat diet with SphK2 inhibitor (HFDi); and **(C)** *Sphk1* and **(D)** *Sphk2* in the hippocampus of the same groups. For panels A-B: n = 5 per group. For panel C-D: n = 5 per group for CD, HFD, and HFDi; n = 4 for CDi. Data are presented as median ± interquartile range (IQR) and analyzed by two-way ART-ANOVA followed by Mann-Whitney multiple comparisons. *Sphk - sphingosine kinase; SphK2i - sphingosine kinase 2 inhibitor.*

© 2025. Vanherle L et al. Published online at http://www.aginganddisease.org/EN/10.14336/AD.2025.0636

SUPPLEMENTARY DATA

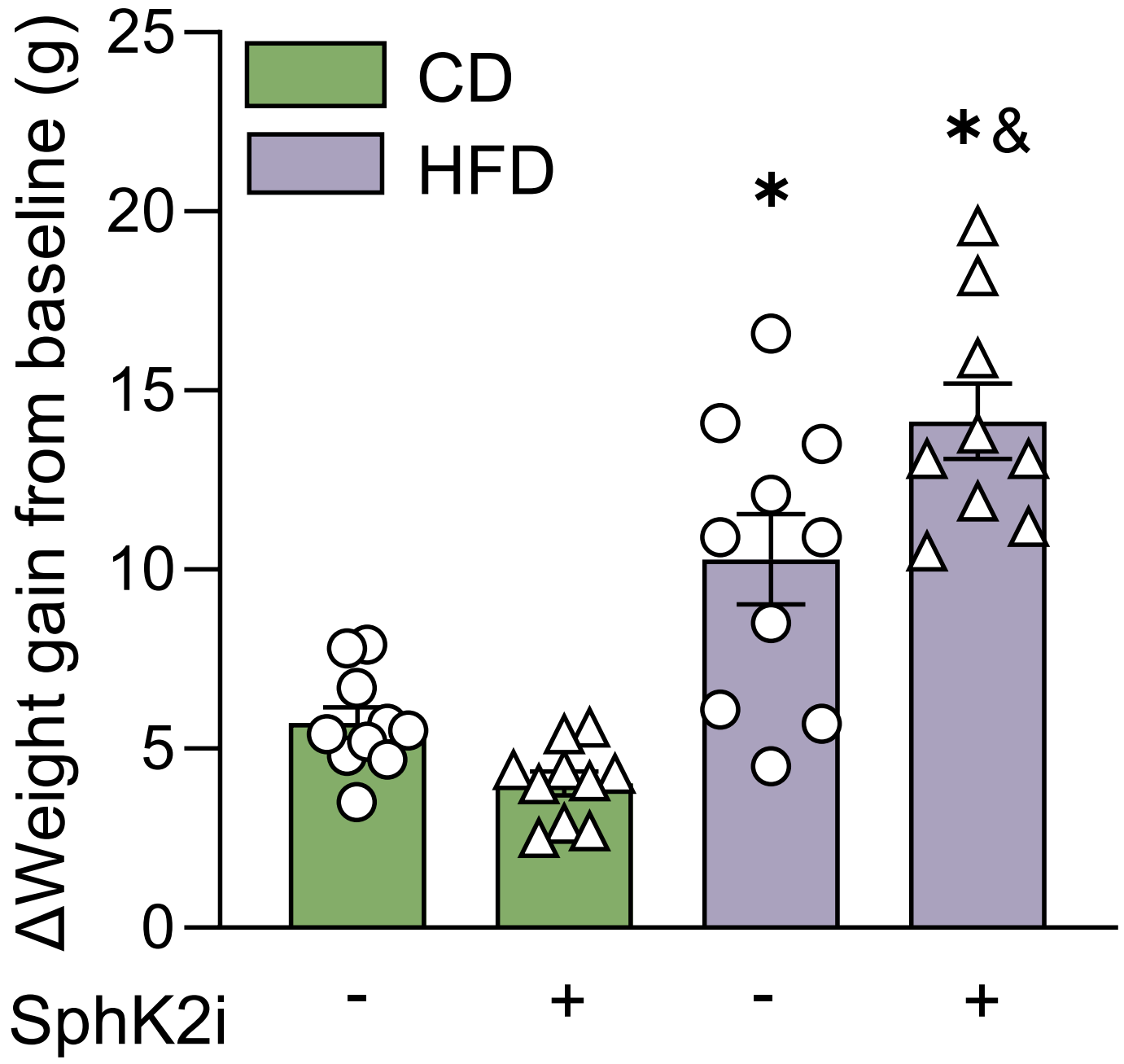

Supplementary Figure 4. SphK2i treatment causes exacerbated weight gain in HFD-fed mice. Body weight gain in mice maintained on control diet (CD), control diet with SphK2 inhibitor (CDi), high-fat diet (HFD), or high-fat diet with SphK2 inhibitor (HFDi). Each point represents an independent biological replicate (n = 10 for CD, CDi, and HFD; n = 9 for HFDi). Data are mean ± SEM and were analyzed by two-way ANOVA followed by LSD multiple comparisons. * $p < 0.05$ *vs*. corresponding CD group; & $p < 0.05$ *vs*. corresponding vehicle group. *SphK2i - sphingosine kinase 2 inhibitor.*

© 2025. Vanherle L et al. Published online at http://www.aginganddisease.org/EN/10.14336/AD.2025.0636

SUPPLEMENTARY DATA

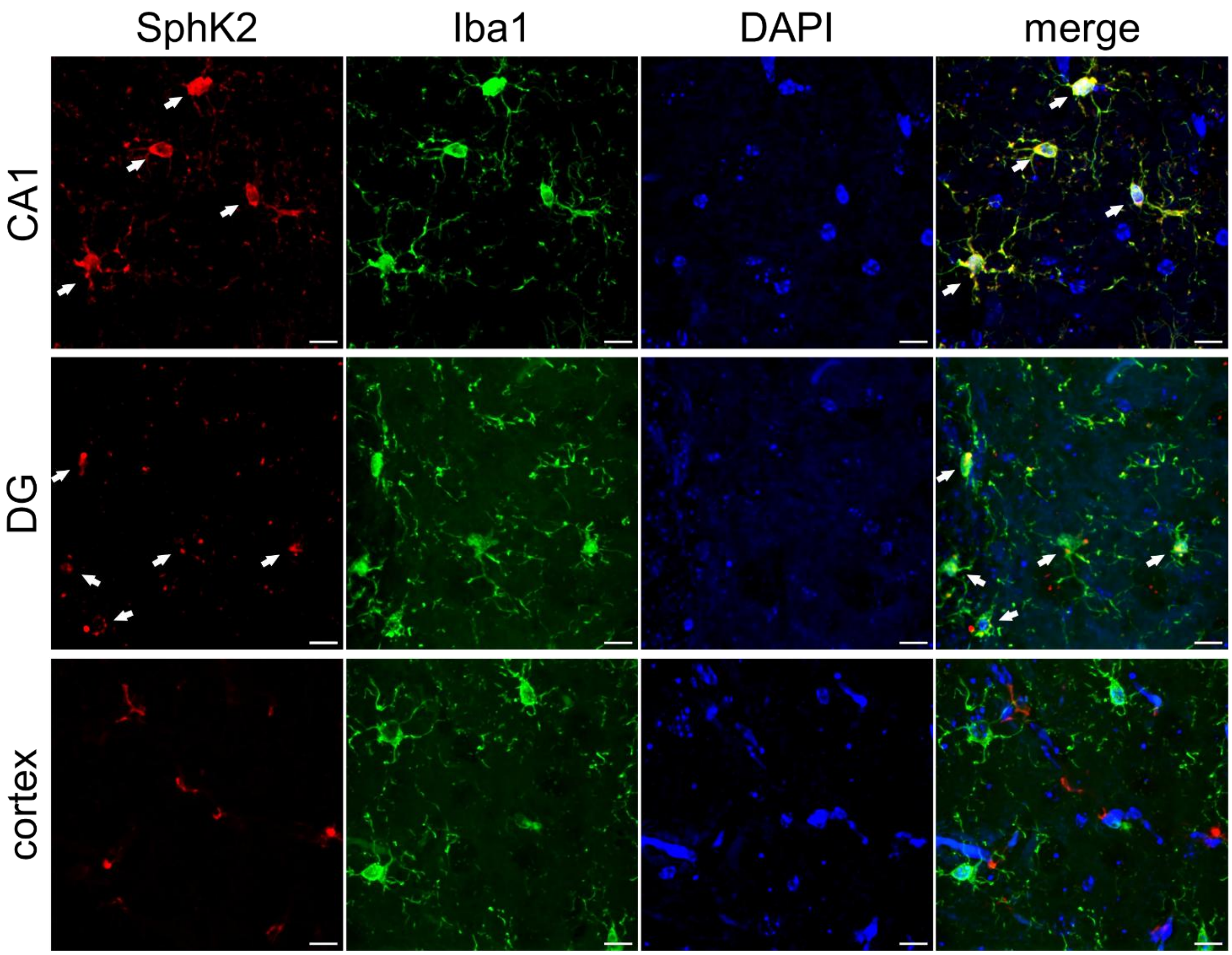

Supplementary Figure 5. Sphingosine kinase 2 co-localizes with microglia in the hippocampus but not in the cortex. Qualitative immunofluorescent-based assessment of sphingosine kinase 2 (SphK2) colocalization with ionized calcium-binding adapter molecule 1 (Iba1) positive microglia. Arrows indicate SphK2-positive microglia cells. Scale bar is 10 µm. CA1 - Cornu Ammonis 1, DG - dentate gyrus.

© 2025. Vanherle L et al. Published online at http://www.aginganddisease.org/EN/10.14336/AD.2025.0636

SUPPLEMENTARY DATA

Supplementary Table 1. Primers used for qRT-PCR.

Target gene	Forward (5' → 3')	Reverse (5' → 3')
Sphk1	GGTGAATGGGCTAATGGAACG	CTGCTCGGTACCCAGCATAGTG
Sphk2	CACGGCGAGTTTGGTTCCTA	CTTCTGGCTTTGGGCGTAGT
Sgpp1	GAGCAACTTGCCGCTCTACTA	GGTCGAGATTCCAGATCCAGAA
Sgpp2	GTTCTCTACGCTGGTGTGTCT	GCAGGGTAGGTCAGAGCAAT
Spgl1	GGTGTATGAGCTTATCTTCCAGC	CTGTTGTTCGATCTTACGTCCA
S1pr1	ATGGTGTCCACTAGCATCCC	CGATGTTCAACTTGCCTGTGTAG
S1pr2	ACAGTCTCCAAAACCAACCACT	ACTCTGAGTATAAGCCGCCCA
S1pr3	CAAGCCTAGCGGGAGAGAAA	ACTGCGGGAAGAGTGTTGAA
Il6	TCTGAAGGACTCTGGCTTTG	GATGGATGCTACCAAACTGGA
Tnfa	TTGACCTCAGCGCTGAGTTG	CCTGTAGCCCACGTCGTAGC
Il1b	GAAGAGCCCATCCTCTGTGA	TTCATCTCGGAGCCTGTAGTG
Cd68	TGTCTGATCTTGCTAGGACCG	GAGAGTAACGGCCTTTTTGTGA
Nos2	GGCAGCCTGTGAGACCTTTG	GCATTGGAAGTGAAGCGTTTC
Rpl14	GGCTTTAGTGGATGGACCCT	ATTGATATCCGCCTTCTCCC

Supplementary Table 2. Statistical table using non-parametric testing for datasets with N<6.

Figure	Parameter	Comparison Test	Statistical Outcome	
2A	Gene expr SphK1 vs. SphK2 Cortex	Mann-Whitney test	SphK1 vs. SphK2 p=0.188	
	Gene expr *Sphk1* vs. *Sphk2* Hippocampus	Mann-Whitney test	SphK1 vs. SphK2 p=0.125	
**Figure**	**Parameter**	**Comparison Test**	**Statistical Outcome**	
2B	*Sphk1* expr CD vs. HFD Cortex	Mann-Whitney test	CD vs. HFD p>0.999	
	Sphk1 expr CD vs. HFD Hippocampus	Mann-Whitney test	CD vs. HFD p=0.222	
2C	*Sphk2* expr CD vs. HFD Cortex	Mann-Whitney test	CD vs. HFD p=0.841	
	Sphk2 expr CD vs. HFD Hippocampus	Mann-Whitney test	CD vs. HFD p=0.056	
**Figure**	**Parameter**	**Comparison Test**	**Statistical Outcome (ANOVA statistic)**	**Statistical Outcome (post hoc)**
2D	Cortical S1P (pmol/mg)	Two-way ART ANOVA	Int: F = 3.500, **p = 0.04**; Drug: F = 0.110, p = 0.744; Diet: F = 6.680, **p = 0.019**	Veh,CD vs. Veh,HFD p=0.056; SphK2i,CD vs. SphK2i,HFD p=0.421, CD,veh vs. CD,SphK2i p=0.151, HFD,veh vs. HFD,SphK2i p=0.460
2E	Hippocampal S1P (pmol/mg)	Two-way ART ANOVA	Int: F = 2.599, p = 0.091; Drug: F = 3.927, p = 0.064; Diet: F = 5.983, **p = 0.026**	Veh,CD vs. Veh,HFD p=0.151; SphK2i,CD vs. SphK2i,HFD p=0.127
3A	Gene expression *Sgpp1* - Cortex	Two-way ART ANOVA	Int: F = 1.235, p = 0.330; Drug: F = 1.306, p = 0.268; Diet: F = 2.752, p = 0.114	-
3B	Gene expression *S1pr1* - Cortex	Two-way ART ANOVA	Int: F = 2.839, p = 0.071; Drug: F = 1.128, p = 0.302; Diet: F = 5.055, **p = 0.037**	Veh,CD vs. Veh,HFD **p=0.032**; SphK2i,CD vs. SphK2i,HFD p=0.841
3C	Gene expression *Sgpp2* - Cortex	Two-way ART ANOVA	Int: F = 6.333, **p = 0.005**; Drug: F= 0.005, p = 0.942; Diet: F = 3.772, p = 0.068	Veh,CD vs. Veh,HFD p=0.548; SphK2i,CD vs. SphK2i,HFD **p=0.008**, CD,veh vs. CD,SphK2i p=0.095, HFD,veh vs. HFD,SphK2i **p=0.032**
3D	Gene expression *Sgpp1* - Hippocampus	Two-way ART ANOVA	Int: F = 2.525, p = 0.097; Drug: F = 1.545, p = 0.231; Diet: F = 7.691, **p = 0.013**	Veh,CD vs. Veh,HFD p=0.310; SphK2i,CD vs. SphK2i,HFD **p=0.032**
3E	Gene expression *S1pr1* - Hippocampus	Two-way ART ANOVA	Int: F = 0.025, p = 0.994; Drug: F = 0.000, p > 0.999; Diet: F = 0.057, p = 0.814	-

© 2025. Vanherle L et al. Published online at http://www.aginganddisease.org/EN/10.14336/AD.2025.0636

SUPPLEMENTARY DATA

3F	Gene expression *Sgpp2* - Hippocampus	Two-way ART ANOVA	Int: F = 1.629, p = 0.225; Drug: F = 5.678, **p = 0.029**; Diet: F = 0.798, p = 0.384	CD,veh vs. CD,SphK2i p=0.286, HFD,veh vs. HFD,SphK2i p=0.095
4A	% microglia (cortex) - morphology	Three-way ART ANOVA	Morphology: F = 206.58, **p < 0.0001**; Diet: F = 0.009, p = 0.925; Treatment: F = 0.001, p = 0.975; Morphology x Diet: F = 83.925, **p < 0.0001**; Morphology x Treatment: F = 80.765, **p < 0.0001**; Diet x Treatment: F = 0.158, p = 0.924; Morphology x Diet x Treatment: F = 45.062, **p < 0.0001**	CD,ram vs. HFD,ram p=0.222; CDi,ram vs. HFDi,ram p=0.905, CD,ram vs. CDi,ram p=0.691; HFD,ram vs. HFDi,ram p=0.413; CD,int vs. HFD,int p=0.095; CDi,int vs. HFDi,int p=0.730, CD,int vs. CDi,int p=0.222; HFD,int vs. HFDi,int p=0.286; CD,amb vs. HFD,amb p=0.206; CDi,amb vs. HFDi,amb p=0.064, CD,amb vs. CDi,amb **p=0.008**; HFD,amb vs. HFDi,amb p=0.413
4B	% microglia (DG) - morphology	Three-way ART ANOVA	Morphology: F = 74.041 **p < 0.0001**; Diet: F = 0.036, p = 0.850; Treatment: F = 0.212, p = 0.647; Morphology x Diet: F = 31.034, **p < 0.0001**; Morphology x Treatment: F = 33.581, **p < 0.0001**; Diet x Treatment: F = 0.098, p = 0.961; Morphology x Diet x Treatment: F = 21.493, **p < 0.0001**	CD,ram vs. HFD,ram p=0.0556; CDi,ram vs. HFDi,ram p=0.191, CD,ram vs. CDi,ram p=0.548; HFD,ram vs. HFDi,ram **p=0.016**; CD,int vs. HFD,int p=0.222; CDi,int vs. HFDi,int p=0.905, CD,int vs. CDi,int p=0.548; HFD,int vs. HFDi,int p=0.191; CD,amb vs. HFD,amb p=0.151; CDi,amb vs. HFDi,amb **p=0.032**, CD,amb vs. CDi,amb p=0.310; HFD,amb vs. HFDi,amb **p=0.032**
4C	% microglia (CA1) - morphology	Three-way ART ANOVA	Morphology: F = 216.6, **p < 0.0001**; Diet: F = 0.006, p = 0.937; Treatment: F = 0.001, p = 0.975; Morphology x Diet: F = 88.257, **p < 0.0001**; Morphology x Treatment: F = 84.168, **p < 0.0001**; Diet x Treatment: F = 0.045, p = 0.987; Morphology x Diet x Treatment: F = 38.156, **p < 0.0001**	CD,ram vs. HFD,ram p=0.841; CDi,ram vs. HFDi,ram p=0.286, CD,ram vs. CDi,ram p=0.691; HFD,ram vs. HFDi,ram p=0.286; CD,int vs. HFD,int p=0.310; CDi,int vs. HFDi,int p=0.191, CD,int vs. CDi,int p=0.691; HFD,int vs. HFDi,int p=0.413; CD,amb vs. HFD,amb p=0.310; CDi,amb vs. HFDi,amb p=0.905, CD,amb vs. CDi,amb p=0.691; HFD,amb vs. HFDi,amb p=0.286
4D	# microglia cells (Cortex)	Two-way ART ANOVA	Int: F = 0.287, p = 0.834; Drug: F = 0.364, p = 0.554; Diet: F = 0.317, p = 0.581	-
4E	# microglia cells (DG)	Two-way ART ANOVA	Int: F = 1.759, p = 0.198; Drug: F = 5.132, **p = 0.037**; Diet: F = 0.006, p = 0.938	CD,veh vs. CD,SphK2i **p=0.048**, HFD,veh vs. HFD,SphK2i p=0.381
4F	# microglia cells (CA1)	Two-way ART ANOVA	Int: F = 3.003, p = 0.064; Drug: F = 4.376, p = 0.052; Diet: F = 4.142, p = 0.058	-
5B	#microglial endpoints (cortex)	Two-way ART ANOVA	Int: F = 4.381, **p = 0.021**; Drug: F = 2.038, p = 0.172; Diet: F = 6.288, **p = 0.023**	Veh,CD vs. Veh,HFD **p=0.008**; SphK2i,CD vs. SphK2i,HFD p=0.730, CD,veh vs. CD,SphK2i p=1.000, HFD,veh vs. HFD,SphK2i p=0.064
5C	Microglial branch length (cortex)	Two-way ART ANOVA	Int: F= 3.523, **p = 0.041**; Drug: F = 4.142, p = 0.058; Diet: F(1,15) = 9.394, **p = 0.007**	Veh,CD vs. Veh,HFD **p=0.032**; SphK2i,CD vs. SphK2i,HFD p=0.191, CD,veh vs. CD,SphK2i p=0.421, HFD,veh vs. HFD,SphK2i p=0.111
5E	#microglial endpoints (DG)	Two-way ART ANOVA	Int: F = 4.219, **p = 0.024**; Drug: F = 0.230, p = 0.638; Diet: F = 9.394, **p = 0.007**	Veh,CD vs. Veh,HFD **p=0.016**; SphK2i,CD vs. SphK2i,HFD p=0.413, CD,veh vs. CD,SphK2i p=0.421, HFD,veh vs. HFD,SphK2i p=0.556
5F	Microglial branch length (DG)	Two-way ART ANOVA	Int: F = 4.775, **p = 0.016**; Drug: F = 0.230, p = 0.638; Diet: F = 14.119, **p = 0.002**	Veh,CD vs. Veh,HFD **p=0.016**; SphK2i,CD vs. SphK2i,HFD p=0.191, CD,veh vs. CD,SphK2i p=0.421, HFD,veh vs. HFD,SphK2i p=0.905
S1A	Protein expression p*S1PR1* - Cortex	Two-way ART ANOVA	Int: F = 2.771, p = 0.075; Drug: F = 0.815, p = 0.379; Diet: F = 6.094, **p = 0.024**	Veh,CD vs. Veh,HFD **p=0.032**; SphK2i,CD vs. SphK2i,HFD p=0.691
S1B	Protein expression p*S1PR1* - Hippocampus	Two-way ART ANOVA	Int: F = 0.483, p = 0.699; Drug: F = 0.654, p = 0.430; Diet: F = 0.057, p = 0.814	-
S2A	Gene expression *S1pr2* - Cortex	Two-way ART ANOVA	Int: F = 2.363, p = 0.110; Drug: F = 1.501, p = 0.236; Diet: F = 0.449, p = 0.511	-

© 2025. Vanherle L et al. Published online at http://www.aginganddisease.org/EN/10.14336/AD.2025.0636

S2B	Gene expression *S1pr3* - Cortex	Two-way ART ANOVA	Int: F = 0.164, p = 0.919; Drug: F = 0.049, p = 0.828; Diet: F = 0.136, p = 0.716	-
S2C	Gene expression *Sgpl1* - Cortex	Two-way ART ANOVA	Int: F = 1.110, p = 0.374; Drug: F = 1.501, p = 0.236; Diet: F = 1.501, p = 0.236	-
S2D	Gene expression *S1pr2* - Hippocampus	Two-way ART ANOVA	Int: F = 2.111, p = 0.142; Drug: F = 2.318, p = 0.146; Diet: F = 0.526, p = 0.478	-
S2E	Gene expression *S1pr3* - Hippocampus	Two-way ART ANOVA	Int: F = 0.046, p = 0.986; Drug: F = 0.159, p = 0.695; Diet: F = 0.025, p = 0.876	-
S2F	Gene expression *Sgpl1* - Hippocampus	Two-way ART ANOVA	Int: F = 0.447, p = 0.723; Drug: F = 0.159, p = 0.695; Diet: F = 0.413, p = 0.529	-
S3A	Gene expression *Sphk1* - Cortex	Two-way ART ANOVA	Int: F = 0.229, p = 0.875; Drug: F = 0.353, p = 0.560; Diet: F = 0.197, p = 0.662	-
S3B	Gene expression *Sphk2* - Cortex	Two-way ART ANOVA	Int: F = 0.621, p = 0.611; Drug: F= 1.943, p = 0.180; Diet: F = 0.449, p = 0.511	-
S3C	Gene expression *Sphk1* - Hippocampus	Two-way ART ANOVA	Int: F = 1.289, p = 0.314; Drug: F = 0.057, p = 0.814; Diet: F = 2.957, p = 0.104	-
S3D	Gene expression Sphk2 - Hippocampus	Two-way ART ANOVA	Int: F = 2.279, p = 0.121; Drug: F = 2.038, p = 0.172; Diet: F = 11.492, **p = 0.004**	-
S4	Weight gain (g)	*See Table S3*		
Table S4	Gene expression *Il6* - Cortex	Two-way ART ANOVA	Int: F = 1.435, p = 0.270; Drug: F = 0.269, p = 0.610; Diet: F = 3.067, p = 0.097	-
	Gene expression *Tnfa* - Cortex	Two-way ART ANOVA	Int: F = 2.330, p = 0.113; Drug: F = 0.136, p = 0.716; Diet: F = 0.449, p = 0.5111	-
	Gene expression *Il1b* - Cortex	Two-way ART ANOVA	Int: F = 2.219, p = 0.126; Drug: F = 0.815, p = 0.379; Diet: F = 1.943, p = 0.180	-
	Gene expression *Cd68* - Cortex	Two-way ART ANOVA	Int: F = 1.259, p = 0.322; Drug: F = 3.406, p = 0.082; Diet: F = 0.815, p = 0.379	-
	Gene expression *Inos* - Cortex	Two-way ART ANOVA	Int: F = 0.698, p = 0.567; Drug: F = 0.449, p = 0.511, Diet: F = 1.501, p = 0.236	-
	Gene expression *Il6* - Hippocampus	Two-way ART ANOVA	Int: F = 1.092, p = 0.383; Drug: F = 0.798, p = 0.384; Diet: F = 1.781, p = 0.200	-
	Gene expression *Tnfa* - Hippocampus	Two-way ART ANOVA	Int: F = 0.358, p = 0.784; Drug: F = 0.958, p = 0.342; Diet: F = 0.000, p > 0.999	-
	Gene expression *Il1b* - Hippocampus	Two-way ART ANOVA	Int: F = 0.396, p = 0.758; Drug: F = 0.314, p = 0.582; Diet: F = 0.654, p = 0.430	-
	Gene expression *Cd68* - Hippocampus	Two-way ART ANOVA	Int: F = 1.312, p = 0.307; Drug: F = 0.526, p = 0.478; Diet: F = 1.545, p = 0.231	-
	Gene expression *Inos* - Hippocampus	Two-way ART ANOVA	Int: F = 1.858, p = 0.180; Drug: F = 0.413, p = 0.529; Diet: F = 3.713, p = 0.071	-

Supplementary Table 3. Statistical table using parametric testing.

© 2025. Vanherle L et al. Published online at http://www.aginganddisease.org/EN/10.14336/AD.2025.0636

SUPPLEMENTARY DATA

Figure	Parameter	Comparison Test	Statistical Outcome	
2A	Gene expr SphK1 vs. SphK2 Cortex	Paired t-test	SphK1 vs. SphK2 p=0.143	
	Gene expr *Sphk1* vs. *Sphk2* Hippocampus	Paired t-test	SphK1 vs. SphK2 p=0.053	
**Figure**	**Parameter**	**Comparison Test**	**Statistical Outcome**	
2B	*Sphk1* expr CD vs. HFD Cortex	Unpaired t-test	CD vs. HFD p=0.881	
	Sphk1 expr CD vs. HFD Hippocampus	Unpaired t-test	CD vs. HFD p=0.236	
2C	*Sphk2* expr CD vs. HFD Cortex	Unpaired t-test	CD vs. HFD p=0.837	
	Sphk2 expr CD vs. HFD Hippocampus	Unpaired t-test	CD vs. HFD p=0.256	
**Figure**	**Parameter**	**Comparison Test**	**Statistical Outcome (ANOVA statistic)**	**Statistical Outcome (post hoc)**
2D	Cortical S1P (pmol/mg)	Two-way ANOVA + Fisher LSD	Int: F(1,16) = 2.725, p = 0.118; Drug: F(1,16) = 0.444, p = 0.515; Diet: F(1,16) = 8.670, **p = 0.010**	Veh,CD vs. Veh,HFD **p=0.005**; SphK2i,CD vs. SphK2i,HFD p=0.374, CD,veh vs. CD,SphK2i p=0.121, HFD,veh vs. HFD,SphK2i p=0.496
2E	Hippocampal S1P (pmol/mg)	Two-way ANOVA + Fisher LSD	Int: F(1,15) = 0.106, p = 0.750; Drug: F(1,15) = 4.033, p = 0.063; Diet: F(1,15) = 3.544, p = 0.079	Veh,CD vs. Veh,HFD p=0.128; SphK2i,CD vs. SphK2i,HFD p=0.301, CD,veh vs. CD,SphK2i p=0.130, HFD,veh vs. HFD,SphK2i p=0.239
3A	*Sgpp1* gene expression - cortex	Two-way ANOVA + Fisher LSD	Int: F(1,16) = 1.004, p = 0.331; Drug: F(1,16) = 0.664, p = 0.427; Diet: F(1,16) = 2.786, p = 0.115	Veh,CD vs. Veh,HFD p=0.077; SphK2i,CD vs. SphK2i,HFD p=0.644, CD,veh vs. CD,SphK2i p=0.217, HFD,veh vs. HFD,SphK2i p=0.896
3B	*S1pr1* gene expression - cortex	Two-way ANOVA + Fisher LSD	Int: F(1,16) = 3.281, p = 0.089; Drug: F(1,16) = 1.200, p = 0.290; Diet: F(1,16) = 6.460, **p = 0.022**	Veh,CD vs. Veh,HFD **p=0.007**; SphK2i,CD vs. SphK2i,HFD p=0.613, CD,veh vs. CD,SphK2i p=0.057, HFD,veh vs. HFD,SphK2i p=0.620
3C	*Sgpp2* gene expression - cortex	Two-way ANOVA + Fisher LSD	Int: F(1,16) = 11.50, **p = 0.004**; Drug: F(1,16) = 0.021, p = 0.888; Diet: F(1,16) = 7.297, **p = 0.016**	Veh,CD vs. Veh,HFD p=0.632; SphK2i,CD vs. SphK2i,HFD **p=0.001**, CD,veh vs. CD,SphK2i **p=0.024**, HFD,veh vs. HFD,SphK2i **p=0.036**
3D	*Sgpp1* gene expression - hippocampus	Two-way ANOVA + Fisher LSD	Int: F(1,15) = 0.800, p = 0.385; Drug: F(1,15) = 0.897, p = 0.359; Diet: F(1,15) = 3.858, p = 0.068	Veh,CD vs. Veh,HFD p=0.448; SphK2i,CD vs. SphK2i,HFD p=0.068, CD,veh vs. CD,SphK2i p=0.972, HFD,veh vs. HFD,SphK2i p=0.200
3E	*S1pr1* gene expression - hippocampus	Two-way ANOVA + Fisher LSD	Int: F(1,15) = 0.007, p = 0.933; Drug: F(1,15) = 0.009, p = 0.927; Diet: F(1,15) = 0.205, p = 0.658	Veh,CD vs. Veh,HFD p=0.793; SphK2i,CD vs. SphK2i,HFD p=0.717, CD,veh vs. CD,SphK2i p=0.996, HFD,veh vs. HFD,SphK2i p=0.898
3F	*Sgpp2* gene expression - hippocampus	Two-way ANOVA + Fisher LSD	Int: F(1,15) = 0.103, p = 0.753; Drug: F(1,15) = 4.638, **p = 0.048**; Diet: F(1,15) = 1.098, p = 0.311	Veh,CD vs. Veh,HFD p=0.604; SphK2i,CD vs. SphK2i,HFD p=0.362, CD,veh vs. CD,SphK2i p=0.110, HFD,veh vs. HFD,SphK2i p=0.201
4A	% microglia (cortex) - morphology	Three-way ANOVA + Fisher LSD	Morphology: F(2,30) = 178.0, **p < 0.0001**; Diet: F(1,15) = 0.265, p = 0.615; Treatment: F(1,15) = 5.109, **p = 0.039**; Morphology x Diet: F(2,30)= 0.892, p = 0.421; Morphology x Treatment: F(2,30) = 0.159, p = 0.854; Diet x Treatment: F(1,15) = 1.295, p = 0.273; Morphology x Diet x Treatment: F(2,30) = 2.398, p = 0.108	CD,ram vs. HFD,ram p=0.056; CDi,ram vs. HFDi,ram p=0.962, CD,ram vs. CDi,ram p=0.531; HFD,ram vs. HFDi,ram p=0.200; CD,int vs. HFD,int **p=0.032**; CDi,int vs. HFDi,int p=0.384, CD,int vs. CDi,int p=0.221; HFD,int vs. HFDi,int p=0.078; CD,amb vs. HFD,amb p=0.796; CDi,amb vs. HFDi,amb p=0.410, CD,amb vs. CDi,amb p=0.546; HFD,amb vs. HFDi,amb p=0.617
4B	% microglia (DG) - morphology	Three-way ANOVA + Fisher LSD	Morphology: F(2,30) = 81.77, **p < 0.0001**; Diet: F(1,15) = 0.293, p = 0.596; Treatment: F(1,15) = 0.072, p = 0.792; Morphology x Diet: F(2,30)= 1.342, p = 0.277; Morphology x Treatment: F(2,30) = 3.051, p = 0.062; Diet x Treatment: F(1,15) = 0.371, p = 0.552; Morphology x Diet x Treatment: F(2,30) = 6.079, **p = 0.006**	CD,ram vs. HFD,ram **p=0.002**; CDi,ram vs. HFDi,ram p=0.198, CD,ram vs. CDi,ram p=0.587; HFD,ram vs. HFDi,ram **p=0.0003**; CD,int vs. HFD,int p=0.165; CDi,int vs. HFDi,int p=0.465, CD,int vs. CDi,int p=0.482; HFD,int vs. HFDi,int p=0.213; CD,amb vs. HFD,amb p=0.066; CDi,amb vs. HFDi,amb **p=0.047**, CD,amb vs. CDi,amb p=0.215; HFD,amb vs. HFDi,amb **p=0.012**
4C	% microglia (CA1) - morphology	Three-way ANOVA + Fisher LSD	Morphology: F(2,30) = 419.5, **p < 0.0001**; Diet: F(1,15) = 2.117, p = 0.166; Treatment: F(1,15) = 0.174, p = 0.682; Morphology x Diet: F(2,30)= 0.622, p = 0.544; Morphology x Treatment: F(2,30) = 0.126, p = 0.882; Diet x Treatment: F(1,15) = 0.376, p = 0.549; Morphology x Diet x Treatment: F(2,30) = 1.091, p = 0.349	CD,ram vs. HFD,ram p=0.682; CDi,ram vs. HFDi,ram p=0.115, CD,ram vs. CDi,ram p=0.472; HFD,ram vs. HFDi,ram p=0.196; CD,int vs. HFD,int p=0.955; CDi,int vs. HFDi,int p=0.181, CD,int vs. CDi,int p=0.692; HFD,int vs. HFDi,int p=0.356; CD,amb vs. HFD,amb p=0.641; CDi,amb vs. HFDi,amb p=0.805, CD,amb vs. CDi,amb p=0.745; HFD,amb vs. HFDi,amb p=0.704
4D	# microglia cells (Cortex)	Two-way ANOVA + Fisher LSD	Int: F(1,15) = 0.543, p = 0.473; Drug: F(1,15) = 0.147, p = 0.707; Diet: F(1,15) = 0.350, p = 0.563	Veh,CD vs. Veh,HFD p=0.348; SphK2i,CD vs. SphK2i,HFD p=0.922, CD,veh vs. CD,SphK2i p=0.427, HFD,veh vs. HFD,SphK2i p=0.812

© 2025. Vanherle L et al. Published online at http://www.aginganddisease.org/EN/10.14336/AD.2025.0636

SUPPLEMENTARY DATA

4E	# microglia cells (DG)	Two-way ANOVA + Fisher LSD	Int: F(1,15) = 0.411, p = 0.531; Drug: F(1,15) = 3.613, p = 0.077; Diet: F(1,15) = 0.002, p = 0.963	Veh,CD vs. Veh,HFD p=0.672; SphK2i,CD vs. SphK2i,HFD p=0.643, CD,veh vs. CD,SphK2i p=0.084, HFD,veh vs. HFD,SphK2i p=0.400
4F	# microglia cells (CA1)	Two-way ANOVA + Fisher LSD	Int: F(1,15) = 0.005, p = 0.944; Drug: F(1,15) = 3.804, p = 0.070; Diet: F(1,15) = 2.882, p = 0.110	Veh,CD vs. Veh,HFD p=0.217; SphK2i,CD vs. SphK2i,HFD p=0.281, CD,veh vs. CD,SphK2i p=0.161, HFD,veh vs. HFD,SphK2i p=0.216
5B	#microglial endpoints (cortex)	Two-way ANOVA + Fisher LSD	Int: F(1,15) = 2.264, p = 0.153; Drug: F(1,15) = 2.656, p = 0.124; Diet: F(1,15) = 6.055, **p = 0.027**	Veh,CD vs. Veh,HFD **p=0.011**; SphK2i,CD vs. SphK2i,HFD p=0.521, CD,veh vs. CD,SphK2i p=0.929, HFD,veh vs. HFD,SphK2i **p=0.048**
5C	Microglial branch length (cortex)	Two-way ANOVA + Fisher LSD	Int: F(1,15) = 0.443, p = 0.516; Drug: F(1,15) = 1.287, p = 0.274; Diet: F(1,15) = 7.888, **p = 0.013**	Veh,CD vs. Veh,HFD **p=0.023**; SphK2i,CD vs. SphK2i,HFD p=0.162, CD,veh vs. CD,SphK2i p=0.737, HFD,veh vs. HFD,SphK2i p=0.235
5E	#microglial endpoints (DG)	Two-way ANOVA + Fisher LSD	Int: F(1,15) = 1.180, p = 0.294; Drug: F(1,15) = 0.676, p = 0.424; Diet: F(1,15) = 9.305, **p = 0.008**	Veh,CD vs. Veh,HFD **p=0.009**; SphK2i,CD vs. SphK2i,HFD p=0.197, CD,veh vs. CD,SphK2i p=0.184, HFD,veh vs. HFD,SphK2i p=0.858
5F	Microglial branch length (DG)	Two-way ANOVA + Fisher LSD	Int: F(1,15) = 0.768, p = 0.395; Drug: F(1,15) = 0.580, p = 0.458; Diet: F(1,15) = 15.13, **p = 0.002**	Veh,CD vs. Veh,HFD **p=0.003**; SphK2i,CD vs. SphK2i,HFD p=0.056, CD,veh vs. CD,SphK2i p=0.251, HFD,veh vs. HFD,SphK2i p=0.938
S1A	pS1PR1 cortex	Two-way ANOVA + Fisher LSD	Int: F(1,16) = 10.47, **p = 0.003**; Treatment: F(1,16) = 1.596, p = 0.215; Diet: F(1,16) = 73.45, **p < 0.0001**	CD vs. HFD **p=0.001**; CDi vs. HFDi **p<0.0001**, CD vs. CDi p=0.166; HFD vs. HFDi **p=0.003**
S1B	pS1PR1 hippocampus	Two-way ANOVA + Fisher LSD	Int: F(1,15) = 0.006, p = 0.938; Treatment: F(1,15) = 1.091, p = 0.313; Diet: F(1,15) = 0.031, p = 0.863	CD vs. HFD p=0.945; CDi vs. HFDi p=0.863, CD vs. CDi p=0.452; HFD vs. HFDi p=0.493
S2A	*S1pr2* gene expression - cortex	Two-way ANOVA + Fisher LSD	Int: F(1,16) = 7.046, **p = 0.017**; Drug: F(1,16) = 1.804, p = 0.198; Diet: F(1,16) = 1.326, p = 0.266	CD vs. HFD p=0.304; CDi vs. HFDi **p=0.016**, CD vs. CDi p=0.368; HFD vs. HFDi **p=0.012**
S2B	*S1pr3* gene expression - cortex	Two-way ANOVA + Fisher LSD	Int: F(1,16) = 0.439, p = 0.517; Drug: F(1,16) = 0.149, p = 0.705; Diet: F(1,16) = 0.093, p = 0.765	CD vs. HFD p=0.803; CDi vs. HFDi p=0.504, CD vs. CDi p=0.469; HFD vs. HFDi p=0.847
S2C	*Sgpl1* gene expression - cortex	Two-way ANOVA + Fisher LSD	Int: F(1,16) = 0.633, p = 0.438; Drug: F(1,16) = 2.821, p = 0.112; Diet: F(1,16) = 1.817, p = 0.197	CD vs. HFD p=0.149; CDi vs. HFDi p=0.701, CD vs. CDi p=0.099; HFD vs. HFDi p=0.541
S2D	*S1pr2* gene expression - hippocampus	Two-way ANOVA + Fisher LSD	Int: F(1,15) = 2.753, p = 0.118; Drug: F(1,15) = 2.585, p = 0.129; Diet: F(1,15) = 1.787, p = 0.201	CD vs. HFD p=0.817; CDi vs. HFDi p=0.057, CD vs. CDi p=0.972; HFD vs. HFDi **p=0.031**
S2E	*S1pr3* gene expression - hippocampus	Two-way ANOVA + Fisher LSD	Int: F(1,15) = 0.274, p = 0.609; Drug: F(1,15) = 0.177, p = 0.680; Diet: F(1,15) = 0.085, p = 0.774	CD vs. HFD p=0.869; CDi vs. HFDi p=0.584, CD vs. CDi p=0.945; HFD vs. HFDi p=0.502
S2F	*Sgpl1* gene expression - hippocampus	Two-way ANOVA + Fisher LSD	Int: F(1,15) = 0.034, p = 0.857; Drug: F(1,15) = 0.544, p = 0.472; Diet: F(1,15) = 0.677, p = 0.424	CD vs. HFD p=0.648; CDi vs. HFDi p=0.500, CD vs. CDi p=0.536; HFD vs. HFDi p=0.692
S3A	*Sphk1* gene expression - cortex	Two-way ANOVA + Fisher LSD	Int: F(1,16) = 0.050, p = 0.825; Drug: F(1,16) = 0.134, p = 0.719; Diet: F(1,16) = 0.169, p = 0.686	CD vs. HFD p=0.896; CDi vs. HFDi p=0.659, CD vs. CDi p=0.682; HFD vs. HFDi p=0.921
S3B	*Sphk2* gene expression - cortex	Two-way ANOVA + Fisher LSD	Int: F(1,16) = 0.316, p = 0.582; Drug: F(1,16) = 1.171, p = 0.295; Diet: F(1,16) = 0.037, p = 0.850	CD vs. HFD p=0.797; CDi vs. HFDi p=0.601, CD vs. CDi p=0.718; HFD vs. HFDi p=0.262
S3C	*Sphk1* gene expression - hippocampus	Two-way ANOVA + Fisher LSD	Int: F(1,15) = 0.259, p = 0.618; Drug: F(1,15) = 0.002, p = 0.964; Diet: F(1,15) = 2.070, p = 0.171	CD vs. HFD p=0.176; CDi vs. HFDi p=0.532, CD vs. CDi p=0.755; HFD vs. HFDi p=0.692
S3D	*Sphk2* gene expression - hippocampus	Two-way ANOVA + Fisher LSD	Int: F(1,15) = 0.077, p = 0.786; Drug: F(1,15) = 1.970, p = 0.181; Diet: F(1,15) = 4.694, **p = 0.047**	CD vs. HFD p=0.189; CDi vs. HFDi p=0.114, CD vs. CDi p=0.266; HFD vs. HFDi p=0.424
S4	Weight gain (g)	Two-way ANOVA + Fisher LSD	Int: F(1,35) = 10.47, **p = 0.003**; Treatment: F(1,35) = 1.596, p = 0.215; Diet: F(1,35) = 73.45, **p < 0.0001**	CD vs. HFD **p=0.001**; CDi vs. HFDi **p<0.0001**, CD vs. CDi p=0.166; HFD vs. HFDi **p=0.003**
Table S4	Gene expression *Il6* - Cortex	Two-way ANOVA + Fisher LSD	Int: F = 0.351, p = 0.562; Drug: F = 0.314, p = 0.583; Diet: F = 3.150, p = 0.095	Veh,CD vs. Veh,HFD p=0.416; SphK2i,CD vs. SphK2i,HFD p=0.114, CD,veh vs. CD,SphK2i p=0.427, HFD,veh vs. HFD,SphK2i p=0.982
	Gene expression *Tnfa* - Cortex	Two-way ANOVA + Fisher LSD	Int: F = 7.582, **p = 0.014**; Drug: F = 0.012, p = 0.916; Diet: F = 0.510, p = 0.485	Veh,CD vs. Veh,HFD p=0.169; SphK2i,CD vs. SphK2i,HFD **p=0.026**, CD,veh vs. CD,SphK2i p=0.080, HFD,veh vs. HFD,SphK2i p=0.060
	Gene expression *Il1b* - Cortex	Two-way ANOVA + Fisher LSD	Int: F = 3.39, p = 0.084; Drug: F = 2.083, p = 0.168; Diet: F = 2.863, p = 0.110	Veh,CD vs. Veh,HFD **p=0.024**; SphK2i,CD vs. SphK2i,HFD p=0.918, CD,veh vs. CD,SphK2i **p=0.034**, HFD,veh vs. HFD,SphK2i p=0.783
	Gene expression *Cd68* - Cortex	Two-way ANOVA + Fisher LSD	Int: F = 0.357, p = 0.557; Drug: F = 2.082, p = 0.168; Diet: F = 0.360, p = 0.557	Veh,CD vs. Veh,HFD p=0.999; SphK2i,CD vs. SphK2i,HFD p=0.410, CD,veh vs. CD,SphK2i p=0.168, HFD,veh vs. HFD,SphK2i p=0.558

© 2025. Vanherle L et al. Published online at http://www.aginganddisease.org/EN/10.14336/AD.2025.0636

	Gene expression *Nos2* - Cortex	Two-way ANOVA + Fisher LSD	Int: F = 0.313, p = 0.584; Drug: F = 0.188, p = 0.670, Diet: F = 1.758, p = 0.204	Veh,CD vs. Veh,HFD p=0.201; SphK2i,CD vs. SphK2i,HFD p=0.595, CD,veh vs. CD,SphK2i p=0.930, HFD,veh vs. HFD,SphK2i p=0.493
	Gene expression *Il6* - Hippocampus	Two-way ANOVA + Fisher LSD	Int: F = 1.195, p = 0.292; Drug: F = 1.145, p = 0.302; Diet: F = 0.956, p = 0.344	Veh,CD vs. Veh,HFD p=0.152; SphK2i,CD vs. SphK2i,HFD p=0.938, CD,veh vs. CD,SphK2i p=0.158, HFD,veh vs. HFD,SphK2i p=0.987
	Gene expression *Tnfa* - Hippocampus	Two-way ANOVA + Fisher LSD	Int: F = 0.012, p = 0.914; Drug: F = 0.390, p = 0.542; Diet: F = 0.048, p = 0.829	Veh,CD vs. Veh,HFD p=0.938; SphK2i,CD vs. SphK2i,HFD p=0.824, CD,veh vs. CD,SphK2i p=0.621, HFD,veh vs. HFD,SphK2i p=0.713
	Gene expression *Il1b* - Hippocampus	Two-way ANOVA + Fisher LSD	Int: F = 0.300, p = 0.592; Drug: F = 0.333, p = 0.573; Diet: F = 0.428, p = 0.523	Veh,CD vs. Veh,HFD p=0.395; SphK2i,CD vs. SphK2i,HFD p=0.943, CD,veh vs. CD,SphK2i p=0.984, HFD,veh vs. HFD,SphK2i p=0.425
	Gene expression *Cd68* - Hippocampus	Two-way ANOVA + Fisher LSD	Int: F = 1.110, p = 0.309; Drug: F = 1.17, p = 0.297; Diet: F = 0.257, p = 0.619	Veh,CD vs. Veh,HFD p=0.696; SphK2i,CD vs. SphK2i,HFD p=0.300, CD,veh vs. CD,SphK2i p=0.985, HFD,veh vs. HFD,SphK2i p=0.141
	Gene expression *Nos2* - Hippocampus	Two-way ANOVA + Fisher LSD	Int: F = 0.668, p = 0.427; Drug: F = 1.252, p = 0.281; Diet: F = 4.599, **p = 0.049**	Veh,CD vs. Veh,HFD p=0.349; SphK2i,CD vs. SphK2i,HFD p=0.060, CD,veh vs. CD,SphK2i p=0.837, HFD,veh vs. HFD,SphK2i p=0.179

Supplementary Table 4. mRNA expression levels of canonical cytokine markers (IL-6, TNF-α, IL-1β), *Cd68* and *Nos2* remain unchanged by diet and treatment in both cortex and hippocampus.

	CD	CDi	HFD	HFDi
**Cortex**				
Il6	0.509 ± 0.298	0.408 ± 0.211	0.622 ± 0.473	0.622 ± 0.257
Tnfa	0.366 ± 0.492	0.512 ± 0.565	0.442 ± 0.477	0.143 ± 0.355
Il1b	0.922 ± 0.319	0.776 ± 0.241	0.741 ± 0.219	0.812 ± 0.193
Cd68	1.067 ± 0.404	0.931 ± 0.199	1.120 ± 0.202	1.048 ± 0.146
Nos2	0.958 ± 0.268	1.011 ± 0.167	0.861 ± 0.160	0.963 ± 0.227
**Hippocampus**				
Il6	0.264 ± 0.253	0.511 ± 0.524	0.463 ± 0.278	0.461 ± 0.246
Tnfa	0.279 ± 0.390	0.062 ± 0.435	0.166 ± 0.428	0.097 ± 0.462
Il1b	0.935 ± 0.441	0.774 ± 0.257	0.678 ± 0.299	0.856 ± 0.359
Cd68	1.014 ± 0.299	1.001 ± 0.199	0.962 ± 0.433	0.886 ± 0.058
Nos2	0.627 ± 0.161	0.610 ± 0.302	0.731 ± 0.336	0.860 ± 0.593

© 2025. Vanherle L et al. Published online at http://www.aginganddisease.org/EN/10.14336/AD.2025.0636
